# Temperature-Dependent Coherent Tunneling across Graphene–Ferritin Biomolecular Junctions

**DOI:** 10.1021/acsami.2c11263

**Published:** 2022-09-23

**Authors:** Nipun Kumar Gupta, Senthil Kumar Karuppannan, Rupali Reddy Pasula, Ayelet Vilan, Jens Martin, Wentao Xu, Esther Maria May, Andrew R. Pike, Hippolyte P. A. G. Astier, Teddy Salim, Sierin Lim, Christian A. Nijhuis

**Affiliations:** † Department of Chemistry, National University of Singapore, 3 Science Drive 3, Singapore 117543, Singapore; ‡ Centre for Advanced 2D Materials, National University of Singapore, 6 Science Drive 2, Singapore 117546, Singapore; § School of Chemical and Biomedical Engineering, Nanyang Technological University, 70 Nanyang Drive, Singapore 637457, Singapore; ∥ Department of Chemical and Biological Physics, Weizmann Institute of Science, Rehovot 76100, Israel; ⊥ Chemistry-School of Natural and Environmental Sciences, Newcastle University, Newcastle upon Tyne NE1 7RU, U.K.; # School of Materials Science and Engineering, Nanyang Technological University, 50 Nanyang Avenue, Singapore 639798, Singapore; ∇ Hybrid Materials for Opto-Electronics Group, Department of Molecules and Materials, MESA+ Institute for Nanotechnology and Centre for Brain-Inspired Nano Systems, Faculty of Science and Technology, University of Twente, P.O. Box 217, 7500 AE Enschede, The Netherlands

**Keywords:** biomolecular electronics, graphene, EGaIn, ferritin, charge transport

## Abstract

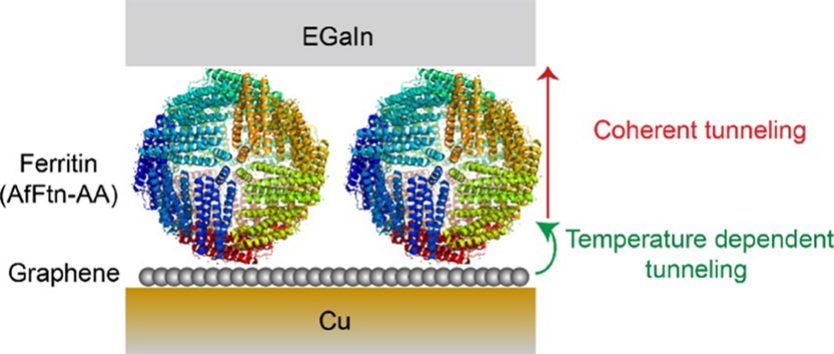

Understanding the mechanisms of charge transport (CT) across biomolecules in solid-state devices is imperative to realize biomolecular electronic devices in a predictive manner. Although it is well-accepted that biomolecule–electrode interactions play an essential role, it is often overlooked. This paper reveals the prominent role of graphene interfaces with Fe-storing proteins in the net CT across their tunnel junctions. Here, ferritin (AfFtn-AA) is adsorbed on the graphene by noncovalent amine–graphene interactions confirmed with Raman spectroscopy. In contrast to junctions with metal electrodes, graphene has a vanishing density of states toward its intrinsic Fermi level (“Dirac point”), which increases away from the Fermi level. Therefore, the amount of charge carriers is highly sensitive to temperature and electrostatic charging (induced doping), as deduced from a detailed analysis of CT as a function of temperature and iron loading. Remarkably, the temperature dependence can be fully explained within the coherent tunneling regime due to excitation of hot carriers. Graphene is not only demonstrated as an alternative platform to study CT across biomolecular tunnel junctions, but it also opens rich possibilities in employing interface electrostatics in tuning CT behavior.

## Introduction

One of the enigmas of charge transport (CT) across biomolecules in biomolecular tunnel junctions is their extremely long coherent distance: over up to tens of nanometers, there is no measurable temperature dependence, in striking contrast to other types of molecules (e.g., polymers or molecular wires) or semiconductors.^1−4^ This weak distance sensitivity implies that the biomolecule–electrode interface is the main variable in CT.^1,5−11^ Still, there is a lack of an in-depth understanding relating the details of such molecule–electrode interfaces to CT efficiencies and characteristics, especially how temperature affects CT. Here, we report that graphene is a viable platform to study CT across proteins in tunnel junctions. We used ferritins (AfFtn-AA, isolated from a hyperthermophilic archaeon *Archaeoglobus fulgidus*) that can be controllably loaded with iron oxide from 500 to 4500 Fe ions per ferritin molecule.^12−14^ We find that charging between the graphene and AfFtn-AA interface depends on the iron oxide loading, which, given the steep energy dispersion of graphene, results in a complex temperature dependency of tunneling rates, while the transmission along AfFtn-AA is temperature-independent. Thus, the temperature-dependent effects are solely caused by interface effects, which is a valuable insight (that may also be applicable in other types of biomolecular tunnel junctions) in the ongoing discussion regarding the mechanisms of CT across biomolecules in solid-state junctions.

The critical role of the interface in CT across (bio)molecular junctions has been long recognized^1,11,15−17^ and includes three main contributions: (i) energy alignment, (ii) molecule–electrode coupling strength (Γ, in eV), and (iii) amount of available carriers. Energy alignment dictates the energy barrier (ε_0_, in eV) between the electrode’s Fermi level and the nearest molecular level, which, in turn, depends on the vacuum position of these levels and interfacial charge rearrangement upon formation of the contacts with the electrodes and associated interface dipoles.^18−23^ This interfacial charging can yield very similar ε_0_ for a given (series of) molecule(s) despite large differences (even as large as 1.5 eV) in the electrode’s Fermi position.^24^ This phenomenon is known as Fermi-level pinning.^25^ Interfacial charging occurs between the electrodes and molecules^18,19,21,23^ and is expected to be even more pronounced for highly charged proteins.^6,26^ In principle, the potential step associated with an interfacial dipole layer is not expected to affect transport because it extends over infinitesimal depth. Nevertheless, strong polarization was suggested to decrease Γ of conjugated molecular wires^24^ and between highly doped Si and bacteriorhodopsin (bR).^6^

The number of charge carriers (*N*_D_) is significantly unknown for large-area molecular junctions due to interface roughness, unknowns in binding densities or ill-controlled area of pressure-sensitive probes.^27^ However, *N*_D_ also reflects electronic considerations;^24,28^ in atomic break junctions, for example, the number of transmission channels correlates with the number of valence electrons for each metal.^29^ In molecular junctions made with a semiconductor electrode, the amount of minority carriers dominates the transport under certain conditions.^30^ In this context, graphene is interesting because it has a limited number of carriers at its Dirac point, but interfacial charging acts as a graphene-dopant varying ε_0_ and consequently *N*_D_.^31,32^

The mechanism of CT across molecular junctions is commonly classified into three generic regimes:^1,4,33^ incoherent tunneling (also called hopping), off-resonance tunneling, and resonant tunneling. In an incoherent process, the charge carrier transiently occupies localized states. Temperature facilitates the propagation from one state to the next, yielding a generic Arrhenius dependence of current density (*J*, in A/cm^*2*^) on temperature, *T* (in K):^1,4^
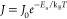
1where *E*_a_ is the activation energy (in eV) and *k*_B_ is the Boltzmann constant (eV/K). In contrast, coherent tunneling processes (regardless of resonance) are independent of the temperature. However, temperature effects become important due to thermal broadening of the Fermi level when ε_0_ is small (ε_0_ < 10*k*_B_*T*)^10,34−37^
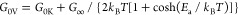
2where *G*_0V_ (in Ω^–1^cm^–2^) is the conductance at 0 V, *G*_0K_ (in Ω^–1^cm^–2^) is the temperature-independent contribution (or saturation conductance), and *G*_∞_ (in *e*VΩ^–1^cm^–2^) is the temperature prefactor. The choice of considering *G*_0V_ instead of *J* is needed for the simplified mathematical term.^35^ Considering that 2 cosh (*E*_a_/*k*_B_*T*)→*E*_a_ ≫ *k*_B_*Te*^*E*_a_/*k*_B_*T*^ implies that eq 2 belongs to a generic Arrhenius-like temperature dependence, though the physical interpretation differs: *E*_a_ is the energy barrier (ε_0_) for near-resonance mechanism and reorganization energy (λ) for hopping.^24,38^ Hopping alone is not expected to show saturation at low temperatures; however, observed CT rates saturate below a threshold temperature due to co-occurring tunneling^38^ or superexchange.^39^

Another experimental observation that helps to discern CT mechanisms is by studying the dependence of distance *d* (in nanometers or distance between the two electrodes) on CT. The sequential nature of hopping implies weak, linear decrease with *d*, which can largely vanish when the hopping step at the molecule–electrode interface becomes the rate-limiting step. In contrast, for coherent tunneling, the value of *J* decays exponentially with *d*:
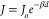
3where *J*_0_ is the pre-exponential factor and β (in nm^–1^) is the tunneling decay coefficient. Coherent tunneling is generally independent of the temperature or activation-less. Generally, ; therefore, off-resonance tunneling is characterized by larger β values than near-resonance cases. Typical values of β in the range of 8–10 nm^–1^ have been reported for junctions derived from saturated *n*-alkanethiols^40^ (off-resonance), but low β values of 2–4 nm^–1^ have been reported for π-conjugated molecular wires^10,41,42^ (near-resonance). It is therefore expected that CT across short molecules is activation-less with high β values for small values of *d* associated with coherent tunneling, but at a certain threshold value for *d*, the mechanism of CT transits to incoherent tunneling characterized by a drop in the value of β and CT exhibits temperature-dependent characteristics of a transition.^43^ This transition has been reported for junctions with conjugated molecular wires^44^ and ferritin.^45^

Biomolecules are known to have extremely low values of β < 2 nm^–1^ over distances >4 nm;^1,3,11^ such an efficient long-distance CT (also referred to as long-range tunneling) seems impossible via coherent processes,^2^ and yet, there are reoccurring examples of temperature-independent CT (azurin,^46^ photosystem-I,^47^ and ferritin^45^). Surprisingly, other proteins show similarly low values of β, yet with temperature-activated CT (bovine serum albumin,^46^ bacteriorhodopsin^46,48^ and E2-LFtn^49^). The mechanisms that enable long-range CT are still poorly understood, and various mechanisms have been proposed, including flickering resonance,^50^ superexchange tunneling,^43^ scattering under strong coupling to the environment,^51^ or where an applied bias shifts molecular orbitals with respect to each other^52^ (similar to intramolecular orbital gating proposed by us^53^).

Globular proteins are, in principle, ideal for studying CT because of their highly symmetrical structure; the protein orientation with respect to the surface normal of the electrodes is not a variable. Ferritin is one such globular protein that consists of 24 identical subunits formed by self-assembly into a symmetrical structure with an external diameter of 12 nm and an 8 nm wide cavity.^13,14^ Ferritin sequesters cytotoxic Fe^II^ and stores the Fe ions in the form of ferrihydrite nanoparticles.^54^ We have previously investigated CT across ferritin (AfFtn-AA; see below for details) junctions immobilized on Au electrodes.^45^ In these junctions, *d* depends on the Fe-ion loading, which can be modulated from 500 to 4500 Fe ions per AfFtn-AA, also denoted as (500Fe)AfFtn-AA or (4500Fe)AfFtn-AA, where the numbers indicate the amount of Fe ions per molecule. In the Au-based junction, CT was temperature-independent for the loading of all measured Fe ions, though we observed a transition from high to low β values (1.30 and 0.28 nm^–1^, respectively) at 3000Fe loading (corresponding to *d* = 7.0 nm). In sharp contrast to the high-to-low β value transition in small molecular wires, which indicates a change in CT from coherent to incoherent tunneling, this transition between two activation-less regimes signifies a transition between two different coherent tunneling regimes (because both CT regimes are activation-less). The origin of this transition is still unclear, but it is important to understand the mechanism of CT in these regimes, which would potentially help to guide future experiments.^2,55^

Here, we report the tunneling behavior of biomolecular junctions where AfFtn-AA is directly adsorbed on graphene supported by Cu. Similar to junctions with AfFtn-AA adsorbed on Au, we observe two CT regimes, namely, a regime with a high β = 1.21 nm^–1^ for 600–3000Fe loadings and a CT regime with a low β = 0.37 nm^–1^ for 3600–4800Fe loadings. However, in contrast to the Au-based analogue junctions, CT is temperature-activated for all Fe loadings, with increasing *E*_a_ for larger Fe loading. The CT-temperature dependence fits well near-resonance thermally excited carriers (eq 2), which identifies *E*_a_ with ε_0_. This energy barrier is very sensitive to Fe loading, varying from 0.1 to 0.45 eV, against prevailing reports on nearly constant energy barrier (or Fermi-level pinning). We attribute both temperature activation and lack of Fermi pinning to the limited density of states in graphene, where AfFtn-AA acts as a dopant, shifting the Dirac point and, thus, the value of *N*_D_. Our study highlights the important role of the electrode–protein interface and suggests that nonmetallic electrodes may reveal new features because they reduce the hybridization of the electrode and molecular states, leaving the emphasis on the molecular character of the junction, helping to discriminate between interfacial effects and the molecular contribution to (bio)molecular tunnel junctions.

## Results and Discussion

### Description of Graphene–AfFtn-AA Junctions

**Figure 1:**
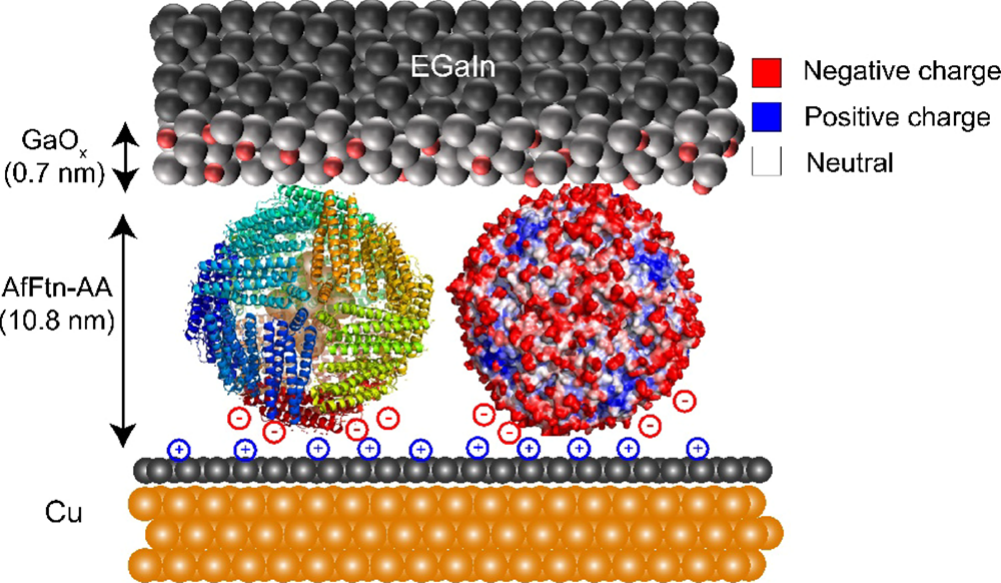
Schematic representation of the Cu//graphene//AfFtn-AA//GaO_*x*_/EGaIn biomolecular tunnel junction, where “//” represents a van der Waals contact and “/” represents the contact between GaO_*x*_ and the bulk eutectic metal alloy. We have used both single-layer graphene and BLG in this study. The left-hand side shows a molecular representation of AfFtn-AA using PyMol. The electrostatic potential map of the AfFtn-AA surface is shown on the right-hand side, where the negatively (red), positively (blue), and neutrally (white) charged amino acids are indicated. The negative charges on the AfFtn-AA (indicated in red) are compensated by positive charges in the Cu//graphene electrode (indicated in blue).

Figure 1 shows a schematic representation of the Cu//graphene//AfFtn-AA//GaO_*x*_/EGaIn tunnel junction, where EGaIn is a liquid metal alloy of Ga and In with a 3:1 ratio (by weight). We used AfFtn-AA (protein database identification code 3KX9) which has high thermal stability (denaturation temperature is 80 °C).^56^ For the CT studies as a function of the iron oxide nanoparticle loading, we used cone-shaped EGaIn tips as the top electrode (contact area of 300–400 μm^2^),^57^ and for the temperature-dependent CT studies, we formed junctions with EGaIn stabilized in through-holes in a microfluidic network (contact area of 1000 μm^2^);^58^ both methods yield junctions with high yields of nonshorting devices with a highly reproducible contact area.^27,59^ The AfFtn-AA isolation and Fe-ion loading and its characterization were carried out via reported procedures^13,45^ (Sections S1–3). As control, we have also investigated CT across junctions with AfFtn-AA with no Fe-ion loading, which is referred to as apo-AfFtn-AA. The outer protein shell of ferritin is negatively charged at pH > 5, and consequently, it has a negative zeta potential.^60^ The presence of the negatively charged shell of AfFtn-AA was confirmed with zeta potential measurements (Section S3). Single-layer and bilayer graphene (BLG) are used in this study, and the graphene (single-layer) was grown on Cu foil by chemical vapor deposition (CVD) following reported procedures^61^ and is described briefly in Section S4. The monolayers of AfFtn-AA were grown on the Cu//graphene substrates in buffered AfFtn-AA solutions for 2 h (see Section S5 for details). Figure 1 shows the structure of AfFtn-AA with iron oxide inside the cavity and the charge density map of the external surface of AfFtn-AA.

### Surface Characterization of AfFtn-AA Monolayers on Graphene

#### Atomic Force Microscopy

**Figure 2:**
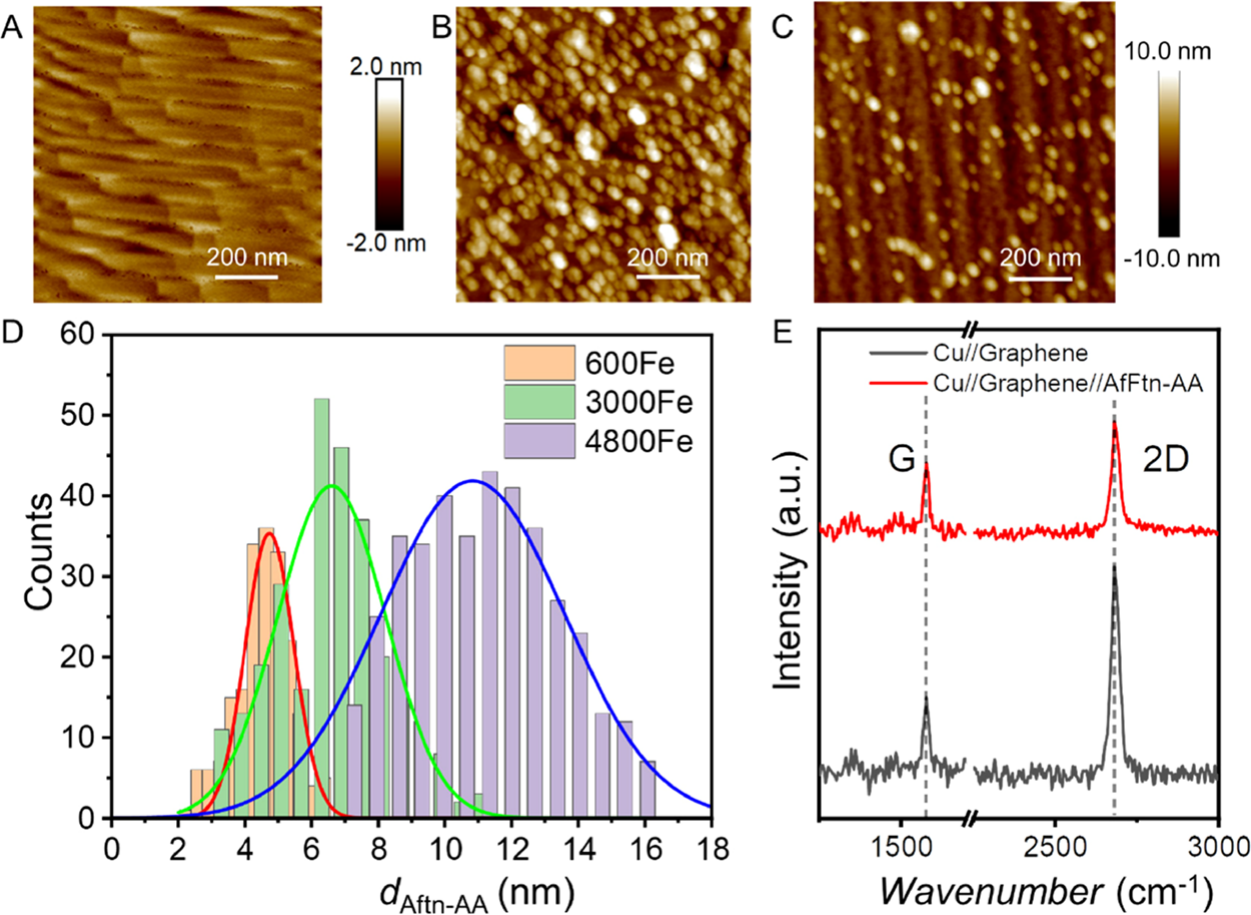
AFM images of (A) Cu//graphene (the scale is presented on the right side), (B) a monolayer of Cu//graphene//(Fe3000)AfFtn-AA, and (C) a sub-monolayer of Cu//graphene//(Fe3000)AfFtn-AA that was utilized to determine the *d*_AfFtn-AA_ [(the scale for (B,C) is presented on the right side of (C)]. (D) Distribution of *d*_AfFtn-AA_ as a function of Fe-ion loading, which is determined from the AFM height profile data. (E) Raman spectra of Cu//graphene (red) and a Cu//graphene//(Fe3000)AfFtn-AA surfaces (black).

We used atomic force microscopy (AFM) to characterize the monolayers of AfFtn-AA immobilized on the Cu//graphene substrates (Section S6). Figure 2A,B shows the AFM images of the Cu//graphene substrates before, and after, deposition of the (3000Fe)AfFtn-AA monolayer, from which we conclude that AfFtn-AA readily forms a dense monolayer on graphene. We reduced the adsorption time of AfFtn-AA to obtain sub-monolayers allowing us to determine the height of individual AfFtn-AA on the graphene surface (*d*_AfFtn-AA_, in nanometers) as a function of Fe-ion loading. Figure 2C shows an AFM image of a sub-monolayer of (Fe3000)AfFtn-AA that was used to determine *d*_AfFtn-AA_ (Figure S2 shows the corresponding AFM line scans). We found that *d*_AfFtn-AA_ = 6.6 ± 0.2 nm (the error represents the 95% confidence level) is comparable to previously reported values of *d*_AfFtn-AA_ of 6.8 ± 0.1 nm for (Fe3000)AfFtn-AA immobilized on Au electrodes.^45^ Similar observations are made for (Fe4800) and (Fe600) AfFtn-AA with *d*_AfFtn-AA_ = 10.8 ± 0.2 and 4.7 ± 0.3 nm, respectively, on graphene, which are remarkably close to *d*_AfFtn-AA_ = 11.1 ± 0.3 and 4.6 ± 0.2 nm for AfFtn-AA monolayers on Au.^45^ Thus, we conclude that the values of *d*_AfFtn-AA_ are within error essentially the same as those obtained from Au-linker surfaces reported in our earlier work.^45^ Therefore, we used the same values of *d*_AfFtn-AA_ below. The *d*_AfFtn-AA_ value for 4500 Fe-ion loading is comparable to the 12.0 nm diameter of AfFtn-AA obtained from crystallographic studies.^62,63^ However, the value of *d*_AfFtn-AA_ from AFM is considerably lower for AfFtn-AA with lower Fe-ion loadings, and therefore, we conclude that partially loaded AfFtn-AA flattens on the Cu//graphene upon adsorption on the surface, while the fully loaded AfFtn-AA retains its globular shape.

#### Raman Spectroscopy

We characterized the monolayers of AfFtn-AA on Cu//graphene with Raman spectroscopy (see Section S7 for details). Figure 2E shows the Raman spectra of Cu//graphene (red) and Cu//graphene//AfFtn-AA (black) surfaces, and Table 1 summarizes all parameters. We found two peaks, namely, the 2*D* peak (at 2680 cm^–1^) and the *G* peak (at 1580 cm^–1^) for both cases. Notably, we did not detect a peak at 1350 cm^–1^ before or after the adsorption of AfFtn-AA, indicating that adsorption of AfFtn-AA does not increase the number of defects in the graphene (see Figure S14). The value of the full width at half-maximum (FWHM) of the 2*D* peak is 29.3 cm^–1^ and hardly changes upon AfFtn-AA adsorption (29.2 cm^–1^). From this observation, we conclude that adsorption of AfFtn-AA does not affect the FWHM or the 2D peak position; thus, AfFtn-AA interacts weakly with the graphene/Cu substrate.^64^ However, the intensity ratio of the 2*D* to *G* peak (*I*_2*D*_/*I_G_*) reduces from 2.8 to 1.5, which also has been observed by others.^65,66^ This observation is attributed to the long-range scattering of electrons (or holes) by adsorbed charged molecules on graphene that reduces the 2*D* band intensity^67^ and thus confirms that charged molecules are adsorbed on the graphene surface. Finally, both spectra lack the *D* peak at ∼1350 cm^–1^ associated with defects in graphene,^68,69^ indicating that our samples are of good quality and not impaired during the adsorption process.

**Table 1:** Raman Characterization of Cu//Graphene and Cu//Graphene//AfFtn-AA

sample	*I*_2*D*_/*I_G_*	FWHM_2*D*_	*G* (cm^–1^)	2*D* (cm^–1^)
Cu//graphene//AfFtn-AA	1.51	29.2	1580	2680
Cu//graphene	2.80	29.3	1580	2680

#### Photoelectron Spectroscopy

The chemical composition of the AfFtn-AA monolayers on graphene was studied with photoelectron spectroscopy (see Section S8). The X-ray photoelectron spectroscopy (XPS) characteristics of AfFtn-AA on the graphene surface are similar to those of our previous study for the AfFtn-AA monolayer on Au,^45^ which reinforces our earlier conclusion that both types of AfFtn-AA monolayers are similar in structure regardless of the substrate.

### CT Measurements

**Figure 3:**
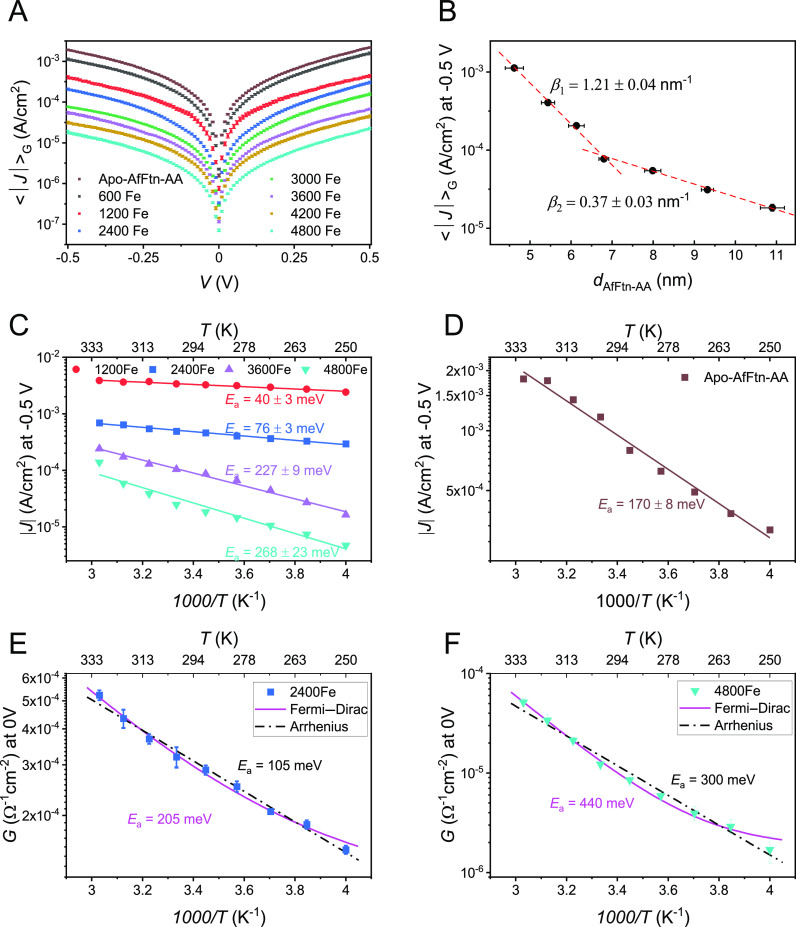
(A) Plots of <log|*J*|>_G_ vs *V* and (B) <log|*J*|>_G_ at −0.5 V vs *d*_AfFtn-AA_ (error bars represent 95% confidence intervals; see Figure S6 for full data distribution) where the dashed lines represent fits to eq 3. Arrhenius plots for *J* at *V* = −0.5 V for representative junctions with AfFtn-AA with different Fe-ion loadings (C) and apo-AfFtn-AA (D) and in terms of *G* around 0 V for (2400Fe)AfFtn-AA (E) and (4800Fe)AfFtn-AA (F). Lines in (C,D) are fits to eq 1 and in (E,F) to eq 2 (magenta) or 4 (dashed black), yielding *E*_a_ values as designated by in-figure labels. Additional data sets and various fitting can be found in Figure S8. *G*(∼0 V) is the slope of current density with respect to the voltage between ±0.02 V (∼5 data points); symbols are the geometric mean of ∼6 repeated voltage loops at each temperature, and error bars represent standard deviation of log*G*(0*V*). Note that the *Y*-axis of E and F spans largely different ranges.

We formed Cu//graphene//AfFtn-AA//GaO_*x*_/EGaIn junctions with cone-shaped tips of EGaIn, recorded large numbers of *J*(*V*) data, and analyzed them with procedures as described in detail elsewhere^57^ (see Section S9). Figure 3A shows the averaged current density, ⟨*J*⟩_*G*_ (derived as 10 ^ ⟨ log |*J*|⟩_*G*_, where ⟨*x*⟩_*G*_ stands for the Gaussian mean), and its 95% confidence levels against applied voltage as a function of the Fe-ion loading. Interestingly, the values of *J* recorded from the Cu//graphene//AfFtn-AA//GaO_*x*_/EGaIn junctions are 1 order of magnitude higher than those values recorded from Au-linker-AfFtn-AA//GaO_*x*_/EGaIn tunnel junctions where the “linker” indicates a thin self-assembled monolayer of 6-mercaptohexanoic acid (with a thickness of 0.9 nm) that was used to anchor AfFtn-AA to the surface.^45^ The nature of binding affects the electrode–molecule coupling and, therefore, the net conductance. In general, physisorbed molecule–electrode contacts have poorer coupling than chemisorbed contacts. Often chemisorbed contacts, however, require linker moieties (usually saturated carbon chains) which reduce the molecule–electrode coupling exponentially with increasing linker length.^70^ The observed increase in the values of *J* indicates that the lack of this linker SAM improves the net electronic coupling between ferritin and graphene despite physisorption.

Figure 3B shows the Gaussian log-average current density, ⟨*J*⟩_*G*_, at *V* = −0.5 V as a function of *d*_AfFtn-AA_ with an abrupt change in slope at *d*_AfFtn-AA_ = 7.0 nm. A fit to eq 3 gives β_1_ = 1.21 ± 0.04 nm^–1^ for *d*_AfFtn-AA_ = 4.5 to 7.0 nm (corresponding to 600Fe to 3000Fe loading) and β_2_ = 0.37 ± 0.03 nm^–1^ for *d*_AfFtn-AA_ = 8.0 to 11.0 nm (corresponding to 3600Fe to 4800Fe loading). The existence of two CT regimes in Cu//graphene//AfFtn-AA is very similar to former findings with Au-based Ferritin junctions, which also showed a transition at ∼3000Fe loading and very close decay values (β_1,Au_ = 1.3 nm^–1^; β_2,Au_ = 0.28 nm^–1^).^45^ Overall, the length decay of CT seems insensitive to the choice of electrode (graphene or Au). However, as discussed later, graphene electrodes make a substantial difference on how temperature affects CT.

### Temperature-Dependent Current–Voltage Measurements

For the temperature-dependent *J*(*V*) measurements, we used EGaIn stabilized in a through-hole in a microfluidic network in polydimethylsiloxane (PDMS) as the top electrode;^58^ see Section S10 for fabrication details. For the bottom electrodes, BLG CVD grown on Si/SiO_2_/Ni/Cu (see Section S11 for details) as a replacement of the Cu-foil to improve the stability of the devices allowing us to conduct *J(V)* measurements over a range of *T* of 250–330 K (see Section S12).

Figure 3C shows the Arrhenius plots for a representative junction of AfFtn-AA with 1200Fe, 2400Fe, 3600Fe, and 4800Fe loadings at *V* = −0.5 V (Supporting Information Figure S8 includes data from additional junctions). Fits to eq 1 are shown by solid lines from which *E*_a_ was determined. Overall, *E*_a_ spans a considerable range from 40 to 270 meV for almost identical proteins, which showed no temperature activation when contacted by Au electrodes.^45^ CT across apo-AfFtn-AA tunnel junctions (Figure 3D; see SI Figure S8 for additional junctions) yielded an intermediate *E*_a_ of 146 meV, roughly a factor of 3 times higher than those junctions with Au electrodes (*E*_a_ = 55 meV).^45^

As mentioned in the Introduction, temperature-activated, Arrhenius-like CT behavior is not necessarily a finger-print of incoherent tunneling (hopping) because a similar dependency can be caused by the temperature effect on the Fermi–Dirac occupation probability of the electrodes (eq 2). Since eq 2 relies on conductance, *G* (cf. current density, *J*), Figure 3E,F reproduces the Arrhenius plots for *G* across AfFtn-AA tunnel junctions for two examples with low and high Fe loadings. The magenta line represents fit to the Fermi–Dirac model (eq 2), while the dashed black line is a fit to the Arrhenius equation in terms of conductance (*G*):
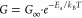
4where the pre-exponential factor, *G*_∞_, relates to the transmission probability at zero barrier (or infinite temperature), similar to *G*_∞_ of eq 2. The main difference in eq 2 compared to eq 4 is the asymptotic saturation to *G*_0K_ as *T* → 0, while the second term of eq 2 is very close to an exponential decay (as in eq 4).

The fit quality of the Fermi–Dirac view (eq 2) is better than the Arrhenius view (eq 4; see *R*^2^ in Figure S10) and yields *E*_a_ that are 1.5–2 times larger than those derived from eq 4. Arguably, the differences are minor in Figure 3E but more pronounced in Figure 3F. Only the Fermi–Dirac view, however, can explain both similarities and differences with previously reported incoherent tunneling across apo-AfFtn-AA junctions immobilized on Au electrodes with *E*_a_^Au^ ≤ 55 meV,^45^ yet with an almost identical distance dependence. Since the molecules are identical for both Au- and BLG-based junctions, the fact that the BLG contact increases the temperature sensitivity relative to that of junctions with Au electrodes supports the Fermi–Dirac view, where the charge transmission along AfFtn-AA is by coherent tunneling, yet the nature of the electrode dictates the Fermi–Dirac population of carriers in the electrode and, in turn, the temperature dependency.

**Figure 4:**
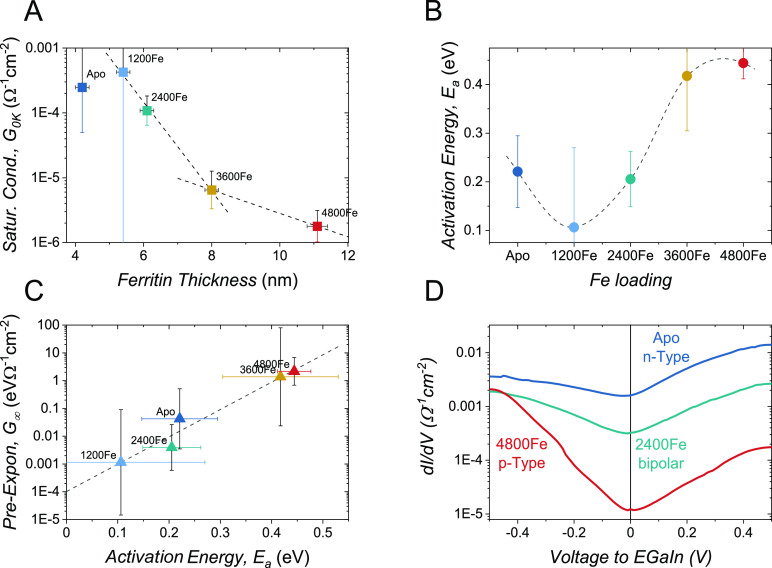
Effect of Fe loading on the conductance, as extracted by fitting *G*(0*V*, *T*) data to the Fermi–Dirac widening model (eq 2) showing (A) saturation conductance, *G*_0K_, against monolayer thickness, with a dual exponential decay marked by black lines; (B) activation energy, *E*_a_; and (C) correlation of the pre-exponential term *G*_∞_ with *E*_a_ showing an exponential trend (dashed line: ln*G*_∞_ ∝ ∼23 · *E*_a_). Error bars are the 95% confidence level in extracted fit parameters; dashed line in (B) is a visual guide. (D) Room-temperature conductance as a function of applied voltage. Color code marks same Fe loading across all panels. Conductance is the first numerical derivative of current w.r.t. voltage. Note that (A–C) refers to the temperature effect at 0 voltage, while panel (D) shows the voltage effect at a constant (room) temperature.

The fit of eq 2 was repeated over five different iron oxide loadings, and the extracted parameters are summarized in Figure 4 (see Supporting Information Figure S7 with Arrhenius plots of current and conductance and fitting to different models and Figure S10 for comparison of parameters extracted by alternative CT-temperature relations). The temperature-independent, saturation conductance (*G*_0K_, Figure 4A) is the easiest to explain as it closely reproduces the length decay of room-temperature *J*(*V*) measurements (Figure 3B). Both show similar values of decay coefficients (β) and a change of slope around a loading of 3000Fe. This similarity reinforces the adequacy of the Fermi–Dirac view (eq 2) in describing CT across Cu//graphene//AfFtn-AA//GaO_*x*_/EGaIn junctions.

### Effect of Fe Loading on Activation Energy

Regardless of fitting to *J*(*T*) (Figure 3C,D) or *G*(*T*) (Figure 3E,F), there is a clear trend of increasing *E*_a_ with Fe loading (span of the *Y*-axis). Figure 4B shows the plot of *E*_a_ against Fe loading. The lowest activation is observed for 1200Fe; its weak temperature dependence implies that this junction does not reach conductance saturation within the measured temperature range (see Figure S7 of the Supporting Information) explaining the considerable uncertainty (error bar) in *G*_0K_ (Figure 4A) of the 1200Fe junction. Around a loading of 3000Fe, *E*_a_ shifts from low to high values (∼0.2 and >0.4 eV, respectively), in agreement with the two CT regimes observed by CT length decay (Figures 3B and 4A).

The Fermi–Dirac model ascribes temperature effects solely to the biomolecule//graphene interface (*E*_a_ is identical to ε_0_ as explained in the Introduction), but, in principle, the Fe loading may alter the core of the biomolecule in the junction. In practice, no change in ε_0_ was observed for AfFtn-AA monolayers on Au with different Fe loadings,^45^ rejecting the possibility that the iron oxide core is responsible for the change in ε_0_. The alternative explanation is a shift in the Fermi energy of BLG upon molecular adsorption, which is quite reasonable in view of the limited density of states of graphene and has been observed experimentally.^31,32^Figure 4B suggests that increasing Fe loading from 1200Fe to 4800Fe shifts the Fermi energy of BLG by ∼0.35 eV. From the Raman spectra recorded for BLG with and without a dense monolayer of (3000Fe)AfFtn-AA shown in Figure S10, we found a shift in the G peak (ΔΩ_G_) of about 5 cm^–1^, which corresponds to a change in *E*_F_ of 0.2 eV (using ΔΩ_G_ = |*E*_F_| × 42 cm^–1^ eV^–1^ following ref^71^) which is reasonably close to the shift in *E*_F_ estimated by the Fermi–Dirac model.

The most surprising parameter is the exponential pre-factor, *G*_∞_ (Figure 4C). Not only it spans 3 orders of magnitude, but also its variation with Fe loading is roughly opposite to that of the saturation conductance (*G*_0K_, Figure 4A; e.g., 4800Fe has the lowest *G*_0K_ but highest *G*_∞_ and vice versa for 1200Fe). This inversion contradicts theory, predicting that both *G*_∞_ and *G*_0K_ are dominated by the coupling strength (Γ)^35^ and that the coupling strength *decreases* exponentially with distance (∼Fe loading; i.e., as observed for *G*_0K_ but opposite to *G*_∞_). A possible clue for the origin of the wide span in *G*_∞_ value comes from the apparent exponential correlation between *G*_∞_ and *E*_a_ (dashed line in Figure 4C), with a high proportionality factor (23 eV^–1^). Prevailing molecular CT models ignore the contacts, assuming that their supply of charge carrier is unlimited and independent of applied voltage and temperature (within measurement limits). Instead, we suggest that the exponential range of *G*_∞_ values emerges from the unique energy dependence of density of states of *graphene* contact (see the next section).

### Proposed Role of Graphene Density of States

**Figure 5:**
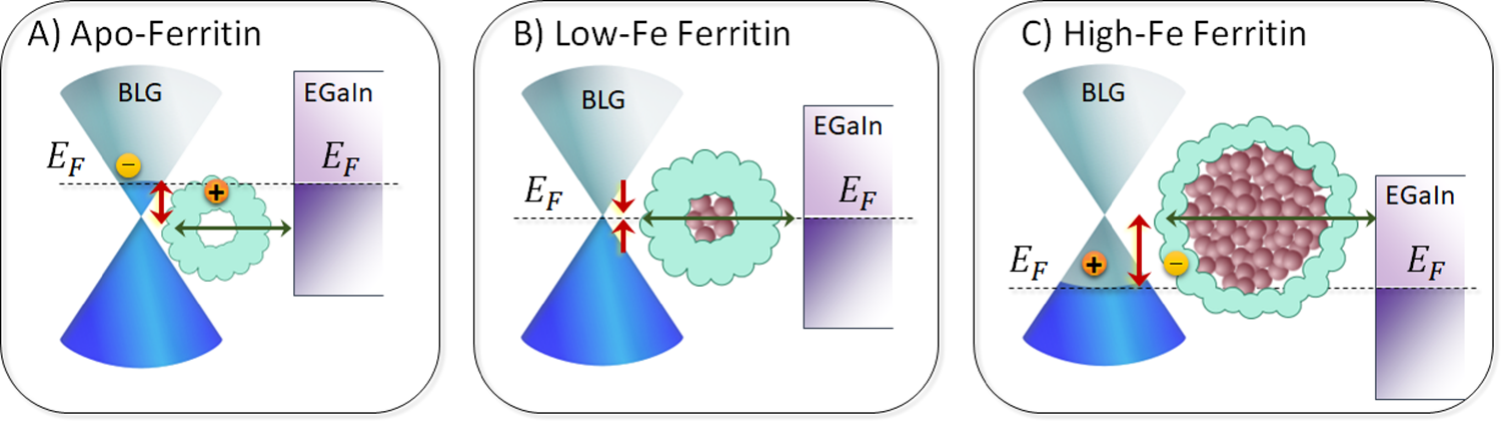
Schematic illustration of energy alignment between BLG (blue-gray dual cones) and (A) apo-AfFtn-AA, (B) AfFtn-AA with small iron oxide, and (C) large iron oxide core. The top-EGaIn contact (purple) is shown without an external bias. Vertical direction represents electron’s energy, and the horizontal direction indicates generally the distance perpendicular to the junction, except for the BLG dual cone representing its energy dispersion with respect to momentum. The horizontal green arrow indicates coherent tunneling, and the vertical red arrow indicates the energy barrier (ε_0_), which varies due to interfacial charging (indicated with the “+” and “–” signs) that shifts the graphene’s Fermi level (*E*_F_). The cone-shaped energy dispersion of graphene implies that small shifts in *E*_F_ translate to exponential changes in the density of available carriers.

Figure 5 summarizes the proposed model derived from our analysis of CT across Cu//graphene//AfFtn-AA//GaO_*x*_/EGaIn junctions based on the Fermi–Dirac view. This model implies that the CT-temperature sensitivity originates from the graphene–AfFtn-AA interface and is not caused by incoherent tunneling across AfFtn-AA (indicated by the horizontal arrow). Upon adsorption of AfFtn-AA, charge rearrangement between AfFtn-AA and BLG is important and depends on the iron oxide loading. The red double arrows indicate the shifts of *E*_F_ in BLG with respect to the charge neutral Dirac point, which defines the barrier height (and associated *E*_a_) as explained earlier. This shift is caused by the charge transfer between the graphene and adsorbed AfFtn-AA as indicated by + and – signs. Electrons move from AfFtn-AA to graphene for apo-AfFtn-AA (Figure 5A), but with increasing Fe loading, the *E*_F_ increasingly shifts downward because of electron transfer from the graphene to AfFtn-AA, resulting in small *E*_a_ values for low Fe loadings (Figure 5B) up to a large *E*_a_ value for high loadings (Figure 5C). With increasing Fe loading, the tunneling distance *d* increases as indicated by the horizontal double arrow.

### Confirmation of Dirac Peak Shift Induced by AfFtn-AA

If AfFtn-AA adsorption induces doping of different types of charge carriers (electrons or holes) depending on Fe loading, we would expect the graphene behavior to change from *n*- to *p*-type.^31,72^ This can be tested by examining the variation of conductance with applied voltage as shown in Figure 4D for three different Fe loadings at room temperature. A junction with 2400Fe loading (mint) is indifferent to the polarity of the applied voltage, while apo-AfFtn-AA (blue) is more conducting at positive voltage and (4800Fe)AfFtn-AA (red) is more conducting at negative bias. This change in the polarity of the rectification agrees with the doping types suggested in Figure 5. The inverted response to bias polarity is observed for all measured junctions and temperature ranges (see Figure S11) under vacuum. Under ambient junctions (see Figure S12), the junctions were largely bipolar, except the extremes apo-AfFtn-AA and (4800Fe)AfFtn-AA which showed a mild *n*- and *p*-type asymmetry, respectively. This is an indirect indication for the great environmental sensitivity of CT across biomolecules.

**Figure 6:**
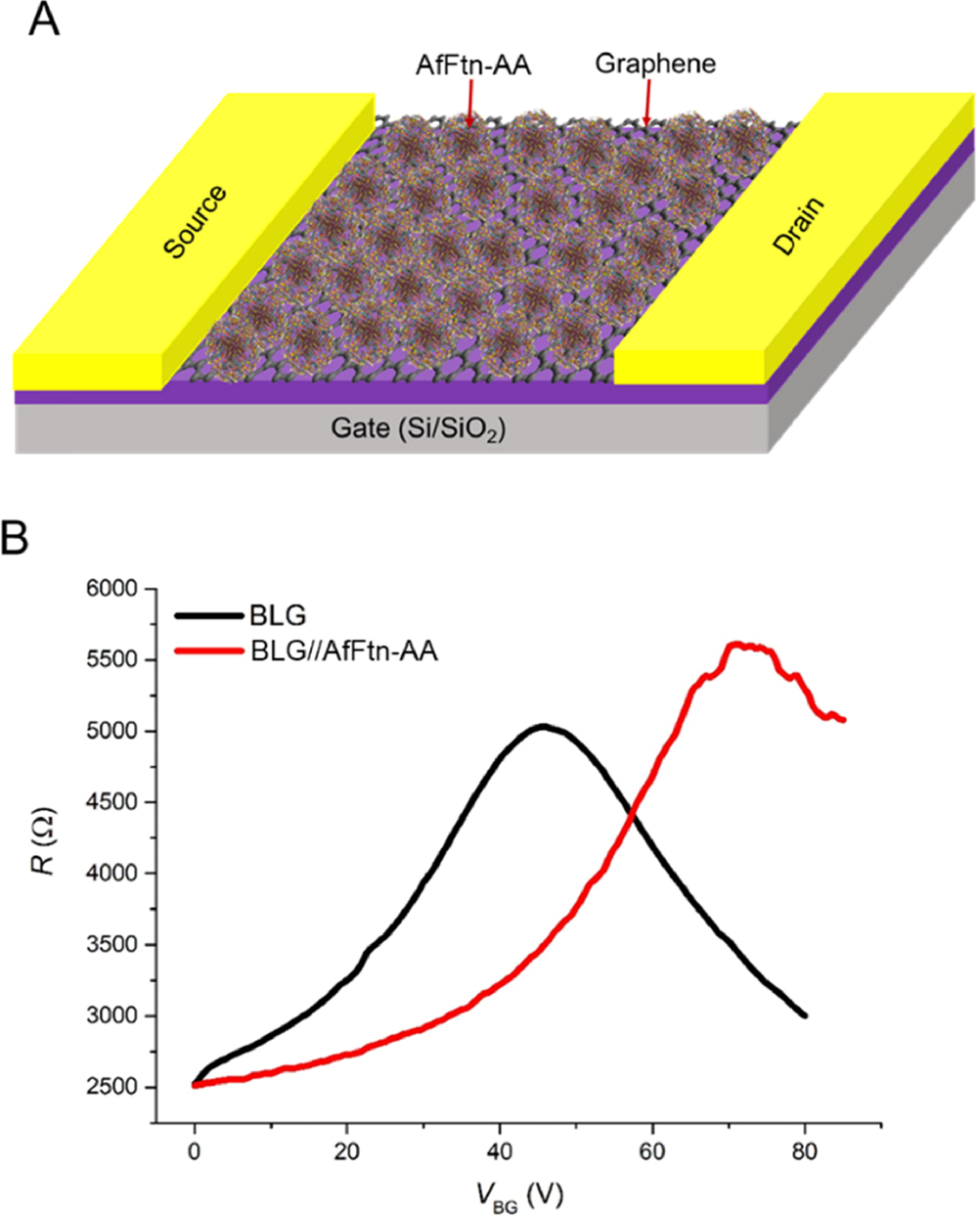
(A) Schematic illustration of a two-terminal GFET device with 3000Fe AfFtn-AA; the device dimensions are 4.5 × 3.1 μm and (B) GFET resistance under vacuum, *R* vs back-gate voltage, and *V*_BG_ curves for a GFET device with (red) and without (black) AfFtn-AA adsorption.

To further confirm the shift in graphene’s Fermi energy at the Cu//Graphene//AfFtn-AA interface, we fabricated GFET devices as detailed in Section S13. Briefly, CVD-BLG (Section S11) was exfoliated using the standard Scotch tape method and then transferred onto the Si/SiO_2_ surface, and the electrical contacts were fabricated using e-beam lithography, followed by thermal deposition of Ti/Au contacts. The GFET device was vacuum annealed and then characterized with Raman spectroscopy and AFM (see Section S13). The Raman spectra of BLG (Section S13, Figure S9) after the adsorption of AfFtn-AA are comparable to the Raman spectra of graphene (see Figure 2) after the adsorption of AfFtn-AA, which suggests that the binding of AfFtn-AA to graphene and BLG is similar in nature. We used BLG-based GFET devices because we also used BLG in the temperature-dependent measurements described in the previous sections. Figure 6A shows the schematics of the GFET device with AfFtn-AA. The resistance (*R*) of the GFET device was measured as a function of back-gate voltage (*V*_BG_) with, and without, AfFtn-AA adsorbed on the graphene (black and red curves in Figure 6B, respectively). The gate voltage of maximal resistance is known as the Dirac peak (*V*_Dirac_), marking the alignment of Fermi level with the Dirac point (“neck” of dual cone in Figure 5). Figure 6B shows that the *V*_Dirac_ peak shifts by 35 V toward a more positive value after AfFtn-AA adsorption compared to clean GFET. This shift indicates a change in the carrier density of BLG caused by molecular-induced doping. This observation is in agreement with previous reports where a highly charged biomolecule was adsorbed on BLG, resulting in a significant shift in *V*_Dirac_.^31,73,74^

## Conclusions

We report that graphene is an interesting bottom-electrode material to immobilize biomolecules and to form biomolecular junctions. AfFtn-AA readily forms a dense monolayer on graphene, allowing us to investigate the mechanism of CT with the EGaIn technique. The Dirac-cone shape of the density of states of graphene implies a finite amount of charge carriers that limits the net CT and is influenced by charge balance at the graphene//AfFtn-AA interface as well as external conditions such as voltage and temperature. The contact-limited carriers can induce significant temperature dependence, although CT across AfFtn-AA per se is activationless as revealed in our earlier work involving CT across AfFtn-AA adsorbed on Au-linker substrates.^45^ In other words, in this work, we were able to decipher the contribution of the “interface” and the “molecule” to the overall temperature dependency of CT across the junction. Our results highlight the importance to investigate CT as a function of the temperature in order to have a detailed understanding of the mechanisms that enable CT. We showed that ferritin adsorption and extent of Fe-loading modulates the type of carriers and their amount as demonstrated for both junction and FET configurations. These results suggest that the properties of the electrode–molecule interface can be utilized as an additional tool to control the temperature-dependent behavior and the rates of CT in molecular junctions. Although in this work we show how graphene affects the temperature dependency of our junctions, potentially by exploiting strain and controlled doping of graphene,^75^ the CT mechanism and rate could be controlled, opening up new ways to control biomolecular electronic devices.

## Supporting Information Available

Experimental details, synthesis, and characterization of AfFtn-AA monolayers on graphene; deposition of graphene; XPS and UPS measurements; statistical analysis of *J*(*V*) measurements; and temperature-dependent *J*(*V*) measurements and GFET measurements (PDF)
