# Supplementary material for: Temperature-Dependent Coherent Tunneling across Graphene–Ferritin Biomolecular Junctions
Source: ACS Appl Mater Interfaces. 2022 Sep 23;14(39):44665–75. doi: 10.1021/acsami.2c11263 (PMC9542697; doi:10.1021/acsami.2c11263)
Supplement: Supplementary file 1 — am2c11263_si_001.pdf [file am2c11263_si_001.pdf]

Supporting Information

Temperature-Dependent Coherent Tunnelling across Graphene-Ferritin Biomolecular Junctions

Nipun Kumar Gupta,[1,2†] *Senthil Kumar Karuppannan,*[1,3†] *Rupali Reddy Pasula,*[4†] Ayelet Vilan,[5*] *Jens Martin*[2], *Wentao Xu*[2], *Esther Maria May,*[6] *Andrew R. Pike,*[7] *Hippolyte P. A. G. Astier,*[1] *Teddy Salim,*[7] *Sierin Lim,*[4*] *and Christian A. Nijhuis*[1,2,8*]

[1]Department of Chemistry, National University of Singapore, 3 Science Drive 3, Singapore 117543, Singapore.

[2]Centre for Advanced 2D Materials, National University of Singapore, 6 Science Drive 2, Singapore 117546, Singapore.

[3]Current address: National Quantum Fabless Foundry (NQFF), Institute of Materials Research and Engineering, 2 Fusionopolis Way, Innovis Building, Singapore 138634, Singapore.

[4]School of Chemical and Biomedical Engineering Nanyang Technological University 70 Nanyang Drive, Singapore 637457, Singapore.

[5]Department of Chemical and Biological Physics, Weizmann Institute of Science, Rehovot 76100, Israel

[6]Chemistry-School of Natural and Environmental Sciences, Newcastle University, Newcastle upon Tyne, NE1 7RU (UK)

[7]School of Materials Science and Engineering, Nanyang Technological University, 50 Nanyang Avenue, Singapore 639798, Singapore.

[8]Hybrid Materials for Opto-Electronics Group, Department of Molecules and Materials, MESA+ Institute for Nanotechnology and Centre for Brain-Inspired Nano Systems, Faculty of Science and Technology, University of Twente, P.O. Box 217, 7500 AE Enschede, The Netherlands.

*Authors to whom correspondence should be addressed: c.a.nijhuis@utwente.nl, slim@ntu.edu.sg, ayelet.vilan@weizmann.ac.il

Section S1. Materials:

We purchased 6 inch silicon wafers with 300 nm SiO_2, 1H,1H,2H,2H-perfluorooctyl trichlorosilane ($Cl_3Si(CH_2)_2(CF_2)_5CF_3$, FOTS) and eutectic gallium-indium (75.5 % Ga and 24.5 % In by weight) from Sigma-Aldrich. Cu foil with a purity of 99.8% was purchased from Alfa Aesar.

Section S2. Preparation and purification of ferritin Aftn-AA with different Fe ion loading:

The Aftn-AA utilised in this study was extracted from a hyper-thermophilic archaeon *Archaeoglobus fulgidus* (PDB ID 3KX9; AfFtn-AA) following a previously reported procedure.[1] *A. fulgidus ferritin* (AfFtn-AA) was produced recombinantly using *E. coli* *B*L21(DE3)C+RIL cells (Stratagene) as the expression host and purified using a combination of thermal and chromatographic method as described elsewhere[2]. The protein quantification was performed using BCA or Bradford assay kit (ThermoFisher Scientific Inc.). The purity of the Aftn-AA preparation was routinely checked using SDS-PAGE.

The Fe loading of Aftn-AA was performed by previously described methods.[3] Apo-Aftn-AA dimers were incubated in freshly prepared $FeSO_4$ solutions in 0.1% HCl for 1 hour at room temperature and then overnight incubated at 4° C. The Aftn-AA were loaded with pre-defined quantity of Fe ion by adding drop-wise the necessary moles of $FeSO_4$ to the dimeric apo-Aftn-AA solution (1 μM Aftn-AA = 500 μg/ mL Aftn-AA). Unbound Fe was removed by buffer exchange with Amicon centrifugal filters (010 kDa MWCO; Millipore). The final amount of Fe loaded was determined using inductively-coupled plasma (ICP) methods.

Section S3. Zeta potential measurements

The zeta potential measurements were carried out with a Malvern zetasizer Nano Z instrument for buffered Aftn-AA to measure the surface electrokinetic potential of Aftn-AAs.

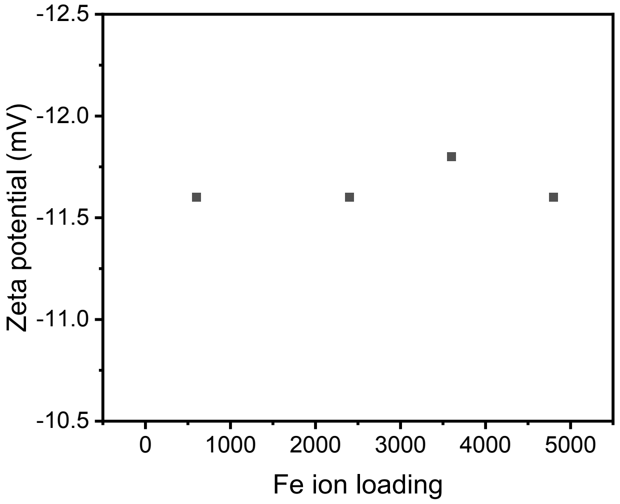

Figure S1: Zeta potential measured for buffered Aftn-AA solutions at pH = 7.5 as a fucntion of the Fe ion loading. The values are the average for 10 independent measurements on each sample with an error of ± 1.0 mV.

Section S4. Preparation of CVD graphene:

We followed a previously reported procedure to obtain single layer graphene on Cu foil using the CVD method.[4] Briefly, a piece of Cu foil (99.8%, Alfa Aesar, No. 13382) was loaded in a quartz tube (semiconductor grade, UFO Labglass), which was first annealed in H_2 with a flow rate of 8 standard cm^2/ minute (sccm) at 1030 °C for 30 min. After annealing, methane gas was introduced into the quartz tube at a flow rate of 16 sccm for 30 min at 1030 °C, and then Cu foil was moved immediately from the heating stage to cool to room temperature. The quality of the SLG on Cu foil was determined by Raman spectroscopy and AFM measurement before use.

Section S5. Self-assembly of Aftn-AA on graphene//Cu:

A 50-100 µL of Aftn-AA solution with 1µM of the buffered Aftn-AA was dropped on a 3 × 3 cm^2 piece of single layer graphene on Cu foil and left for 2 h, after which the substrates were washed gently with deionised water and dried under a flow of N_2 gas.

Section S6. Atomic force microscopy (AFM):

The AFM images of the graphene with and without Aftn-AA were obtained by Bruker dimension Fastscan AFM with tapping mode tips (FASTSCAN-A, resonant frequency: 1.4 MHz, force constant: 18 N/m). We used a Nanoscope analysis (version 1.8) software to extract line profiles (Fig. S2) and determine the height of Aftn-AA on the surface.

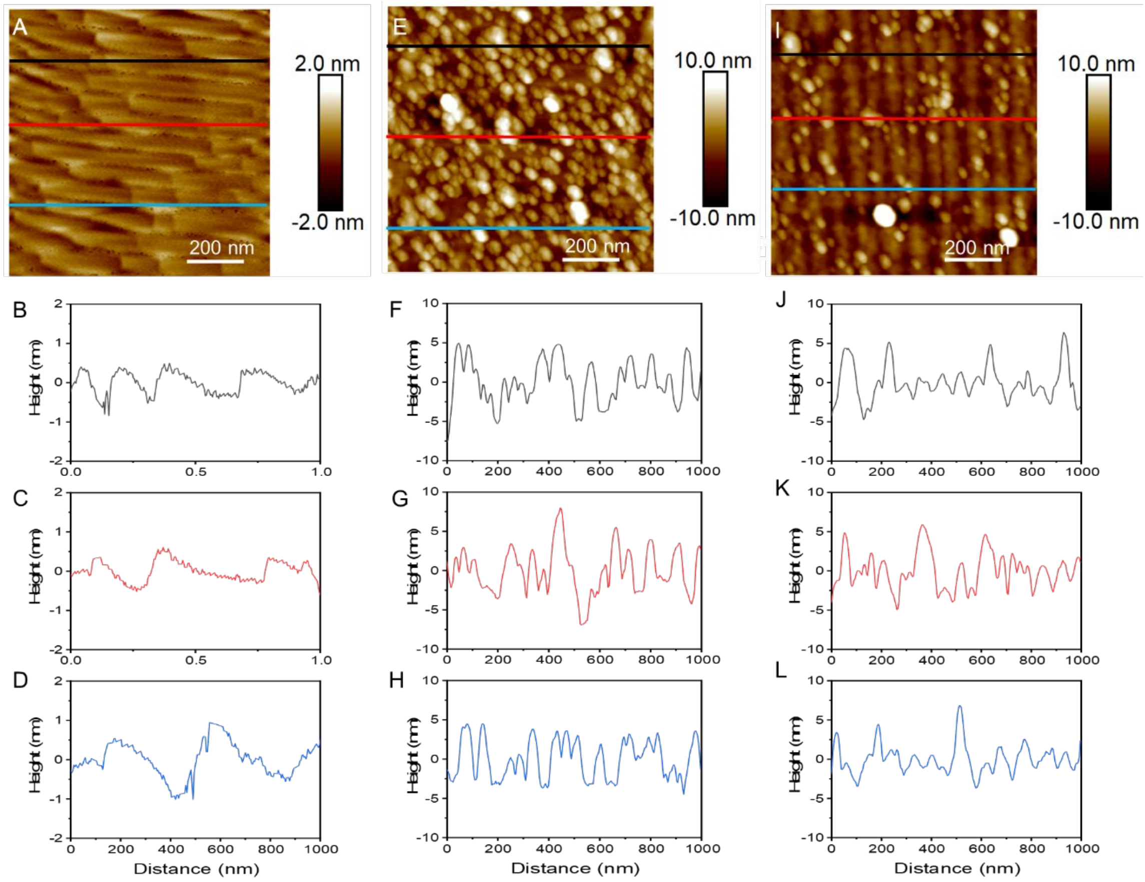

Figure S2: (A) AFM image of a Cu//graphene substrate and the height profile (B, C and D) obtained from the corresponding line scans as indicated on the AFM image in panel A. (E) AFM images of a monolayer of 3000Fe Aftn-AA on Cu//graphene and I) that of a sub-monolayer of Aftn-AA. The corresponding height profiles are given in panels F-H and J-L, respectively.

Section S7. Raman Spectroscopy:

The CVD graphene with and without Aftn-AA was characterised with a WITEC (alpha300 R) Raman system equipped with a 532 nm laser.

Section S8. Photoelectron spectroscopy:

We used an AXIS Supra XPS instrument (Kratos Analytical Ltd, UK) to characterise the chemical composition for the monolayers of Aftn-AA with different Fe ion loadings on Cu//graphene and we followed previously reported procedures to record and analyse the XPS data.[5] The peak assignments are described in the main text, and the binding energies of measured elements are summarised in Table S1. The XPS characteristics of a monolayer of Aftn-AA on the graphene surface are similar to that of our previous XPS investigation of a monolayer of Aftn-AA on a Au surface[1]. Figure S3 shows the XPS spectra for Fe $2p_{3/2}$ for apo-Aftn-AA and different Fe loadings (1200Fe, 2400, and 4800Fe). The polyphasic iron oxide nanoparticles formed in the core of the Aftn-AA consist of haematite (α-Fe_2O_3), maghemite (γ-Fe_2O_3) and iron oxyhydride (α-FeOOH) iron oxides.[6,7] The α-FeOOH exhibits a broad peak at 711.9 eV and this peak shifts to higher binding energy as a function of Fe loading.[6] γ-Fe_2O_3 consists of a single peak at 710.8 eV which shifts to higher binding energy

as a function of loading. Finally, α-Fe_2O_3 is reported at 710.6 eV and is prevalent in higher Fe loadings than in lower Fe loadings.[7]

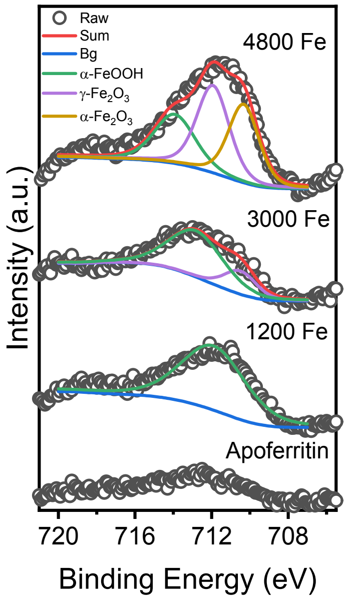

Figure S3: Fe $2p_{3/2}$ spectra recorded on Aftn-AA monolayers with different Fe loadings and apo-Aftn-AA.

Figure S4 shows the XPS spectra for C 1*s*, O 1*s*, Cu 2*p* and N 1*s*. Briefly, the C 1*s* was fitted to three peaks, corresponding to C=C from the protein and graphene and C–H at 284.7 eV (labelled as peak 1), C–N and C–O at 286.1 eV (labelled as peak 2) and NHC=O and –COOH groups at 288.3 eV (labelled as peak 3). The peak at 932.4 eV corresponds to Cu 2*p*. The N 1*s* spectra were fitted to one peak at 400.2 eV which is attributed to C-N. The O 1*s* was fitted to three peaks which are at 531.6, 533.2 eV and 530.2 eV corresponding to –C–OH (peak 1), –O–C=O groups (peak 2) and *α*-FeOOH (peak 3)[8]. The peak at 530.2 eV due to α-FeOOH is only observed at higher Fe loadings.[8]

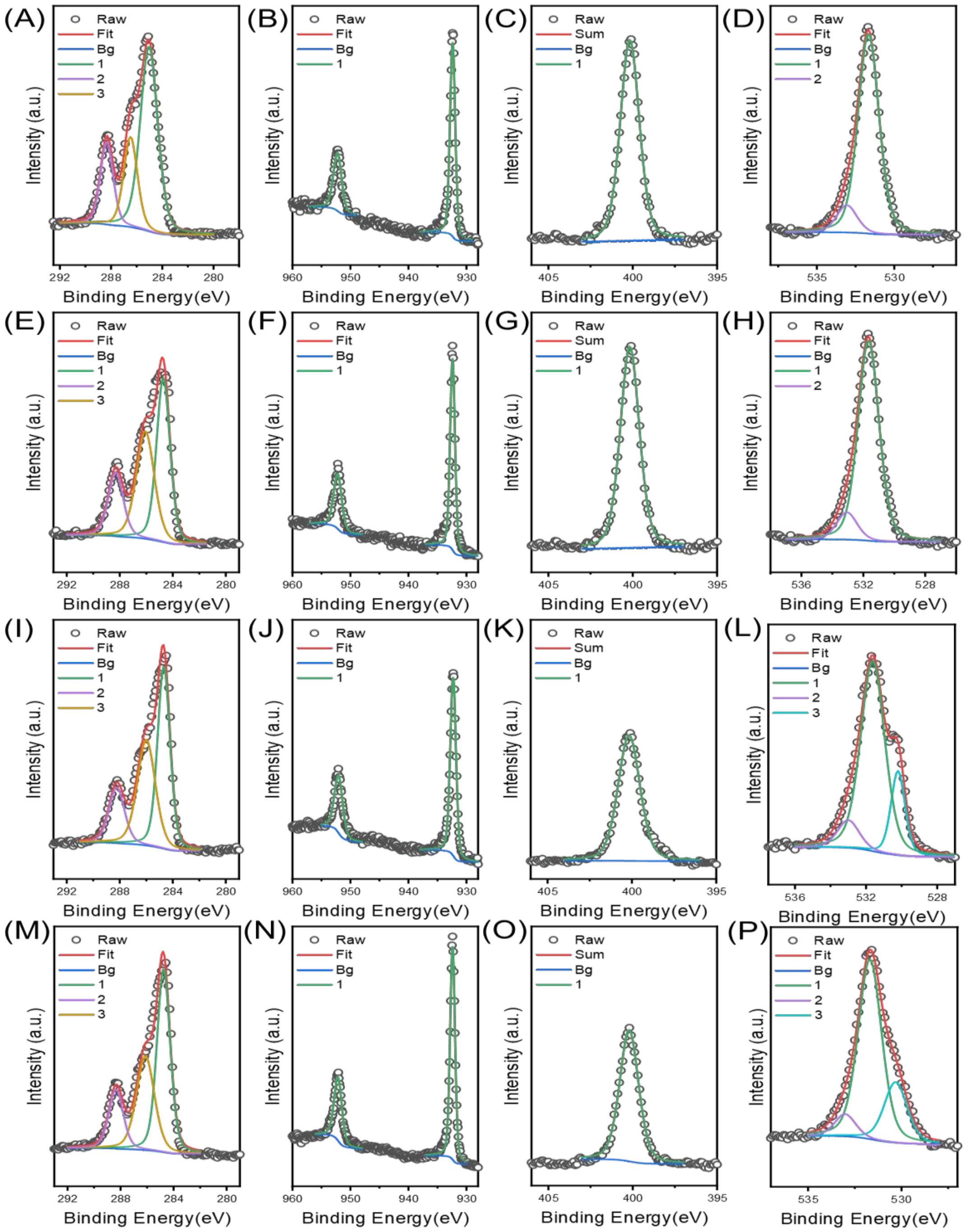

Figure S4: XPS spectra of (A) C 1*s*, (B) Cu 2*p*, (C) O 1*s* and (D) N 1*s* for Cu//graphene//apo-Aftn-AA; XPS spectra of (E) C 1*s*, (F) Cu 2*p*, (G) O 1*s* and (H) N 1*s* for Cu//graphene//1200 Fe Aftn-AA; XPS spectra of (I) C 1*s*, (J) Cu 2*p*, (K) O 1*s* and (L) N 1*s*

for Cu//graphene//3000 Fe Aftn-AA; XPS spectra of (M) C 1*s*, (N) Cu 2*p*, (O) O 1*s* and (P) N 1*s* for Cu//graphene//4800 Fe Aftn-AA.

Table S1: Binding energy of Fe 2*p*, O1*s*, N1*s*, and C1*s* signals of different iron loaded Aftn-AA monolayers

Sample	Fe $2p_{3/2}$ (eV)	C 1*s* (eV)	O 1*s* (eV)	Cu 2*p* (eV)	N 1*s* (eV)
Apo-Aftn-AA	-	285.0, 286.5, 288.3	531.7, 533.1	932.4	400.2
1200Fe	711.9	284.7, 286.1, 288.3	531.5, 532.2	932.4	400.2
3000Fe	710.5, 712.8	284.7, 286.0, 288.2	530.2, 531.6, 533.0	932.3	400.2
4800Fe	710.3, 711.9, 713.9	284.8, 286.1, 288.3	530.3, 531.7, 533.0	932.4	400.2

Section S9. The Cu//graphene//Aftn-AA//GaO_x/EGaIn junction formation and *J*(*V*) measurements:

The fabrication of the cone-shaped tip of EGaIn and the formation of electrical contacts with the monolayers of Aftn-AA with various Fe ion loading were carried out using reported procedures described elsewhere in detail.[9] The Cu foil with protein monolayers was grounded, and an external bias was applied via the cone-shaped EGaIn. A Keithley 6340 source meter was utilised to collect the *J*(*V*) data. The bias was changed from 0 V→ 0.5 V → 0 V → -0.5 V → 0 V. Three scans were recorded for each junction to ensure the junction was stable and these scans were included in *J*(*V*) data analysis. For stable junctions, we then recorded 20 traces which were used in our analysis. Shorts and open circuits were not used for data analysis. A summary of the *J*(*V*) data and junction yields are shown in Table S3.

We followed the procedure for statistical analysis of the junction data as reported before.[9] The values of $\log_{10}|J|$ measured at each bias were plotted in a histogram, as shown in Fig. S6, to which we then fitted a Gaussian to determine the Gaussian log-average value of

log|*J*|, < log|*J*|>$_G$, the log-standard deviation of *J*, $\sigma_{log,}$ and the 95% confidence bands. The <$\log_{10}$|*J*|>$_G$, σ_{log} values are shown in Table S3.

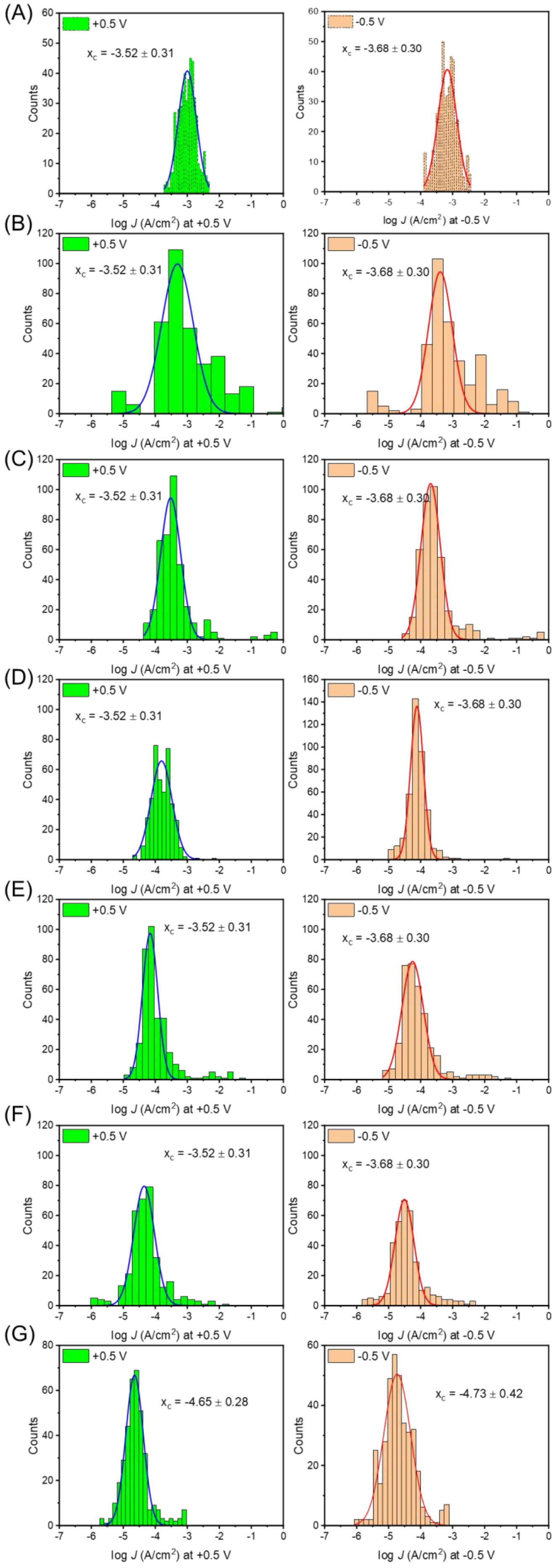
(A)
+0.5 V
x_c = -3.52 ± 0.31
-0.5 V
x_c = -3.68 ± 0.30
(B)
+0.5 V
x_c = -3.52 ± 0.31
-0.5 V
x_c = -3.68 ± 0.30
(C)
+0.5 V
x_c = -3.52 ± 0.31
-0.5 V
x_c = -3.68 ± 0.30
(D)
+0.5 V
x_c = -3.52 ± 0.31
-0.5 V
x_c = -3.68 ± 0.30
(E)
+0.5 V
x_c = -3.52 ± 0.31
-0.5 V
x_c = -3.68 ± 0.30
(F)
+0.5 V
x_c = -3.52 ± 0.31
-0.5 V
x_c = -3.68 ± 0.30
(G)
+0.5 V
x_c = -4.65 ± 0.28
-0.5 V
x_c = -4.73 ± 0.42
Counts
log J (A/cm2) at +0.5 V
log J (A/cm2) at -0.5 V

Figure S5: Histograms of log|*J*| obtained from Cu//graphene//Aftn-AA//GaO_x/EGaIn junctions measured at ±0.5 V for Aftn-AA with Fe loading of A) 500Fe, B) 1200Fe, C) 2400Fe, D) 3000Fe, E) 3600Fe, F) 4200Fe and G) 4800Fe.

Table S2: Summary of the junction statistics.

Molecule	$d_{Aftn-AA}$†	<log|*J*|>$_G$ at +0.5 V (A/cm^2)	σ_{log} at +0.5 V	<log|*J*|>$_G$ at -0.5 V (A/cm^2)	σ_{log} at -0.5 V	Total number of Junctions	Stable junctions	Yield
Apo-Aftn-AA	4.2 ± 0.2	-2.66	0.02	-2.72	0.02	26	22	84.6
600Fe	4.6 ± 0.2	-2.80	0.03	-2.95	0.03	28	23	82.1
1200Fe	5.4 ± 0.2	-3.36	0.04	-3.39	0.04	30	24	80.0
2400Fe	6.1 ± 0.2	-3.52	0.03	-3.69	0.03	24	20	83.3
3000Fe	6.8 ± 0.1	-3.80	0.03	-4.11	0.02	25	20	80.0
3600Fe	8.0 ± 0.2	-4.17	0.02	-4.26	0.03	23	18	78.3
4200Fe	9.3 ± 0.2	-4.35	0.03	-4.51	0.03	22	18	81.8
4800Fe	11.1 ± 0.3	-4.65	0.03	-4.74	0.04	26	20	76.9

†Taken from ref.[1]

Section S10. Fabrication of EGaIn stabilised in a through-hole in PDMS to form top-contacts:

We fabricated top-electrodes with the EGaIn stabilised in a through-hole in PDMS using a previously reported method.[10] Briefly, the moulds were fabricated by a standard two-step photolithography process (see ref 6 for the detailed fabrication procedure). The mould was treated with FOTS to reduce the adhesion of the PDMS with the mould. The filling of the through-hole with EGaIn was monitored by measuring the resistance between the EGaIn and the ITO surface onto which the microfluidic network was placed. Once the though-hole was filled and the EGaIn contacted the ITO (which was also monitored visually), the top electrode was removed from the ITO surface and placed on the monolayer to complete the junction.

Section S11. CVD graphene growth on Cu/Ni deposited on Si wafer:

We followed a previously reported procedure to obtain graphene on $Si/SiO_2/Ni/Cu$ substrate using the CVD method.[11] Briefly, we deposited 100 nm Ni followed by 1000 nm Cu on a 4 inch Si wafer with 300 nm SiO_2 substrate using ultra-high vacuum sputtering (AJA ATC-2200 magnetron sputtering systems). The substrate was kept at 300 K during Ni/Cu metal deposition, the sputtering rate of Ni was about 0.78 nm/s, and the rate of Cu is 0.82 nm/s. The substrate with Cu-Ni alloy deposited on it was placed in a CVD furnace. First, the temperature rose to 650 °C at a rate of 100 °C/min in a H_2 atmosphere. The pressure was maintained at 15 mbar, and then 20 sccm H_2 and 5 sccm methane gas were introduced into the chambe, and the gas flow and temperature was maintained for 5 mins to facilitate the growth of graphene. Finally, the furnace was cooled down to room temperature under a 20 sccm H_2 and 5 sccm methane gas flow. The CVD graphene grown on the substrate was characterised with Raman and AFM. Figure S7 shows AFM images of a black $Si/SiO_2/Ni/Cu$//Graphene substrate (Fig. S7A) and a $Si/SiO_2/Ni/Cu$//Graphene//3000Fe Aftn-AA substrate (Fig. S7B). These substrates have a higher roughness of 4.28 nm over 1×1 μm^2 than the Cu//graphene surfaces of 3.18 nm over 1×1 μm^2 (Fig. 2). We measured Raman spectra of the CVD graphene grown on $Si/SiO_2/Ni/Cu$ substrate before and after Aftn-AA adsorption (Fig. S7C-D). The observed full width at the half maximum of the 2*D* peak (~60 cm^{-1}) and the observed *D* peak around 1200 cm^{-1} confirms the presence of the bilayer graphene with surface defective sp^2 carbon atoms with the intensity of the *D* peak to the *G* peak ratio of <0.4 indicating that BLG forms with a lesser defect density on Ni surface.[12–14] The 2*D* peak shifts (~12 cm^{-1}) to a lower wavenumber after ferritin adsorption on the graphene surface, indicating that highly charged Aftn-AA molecule induces charge transfer across the interface[15].

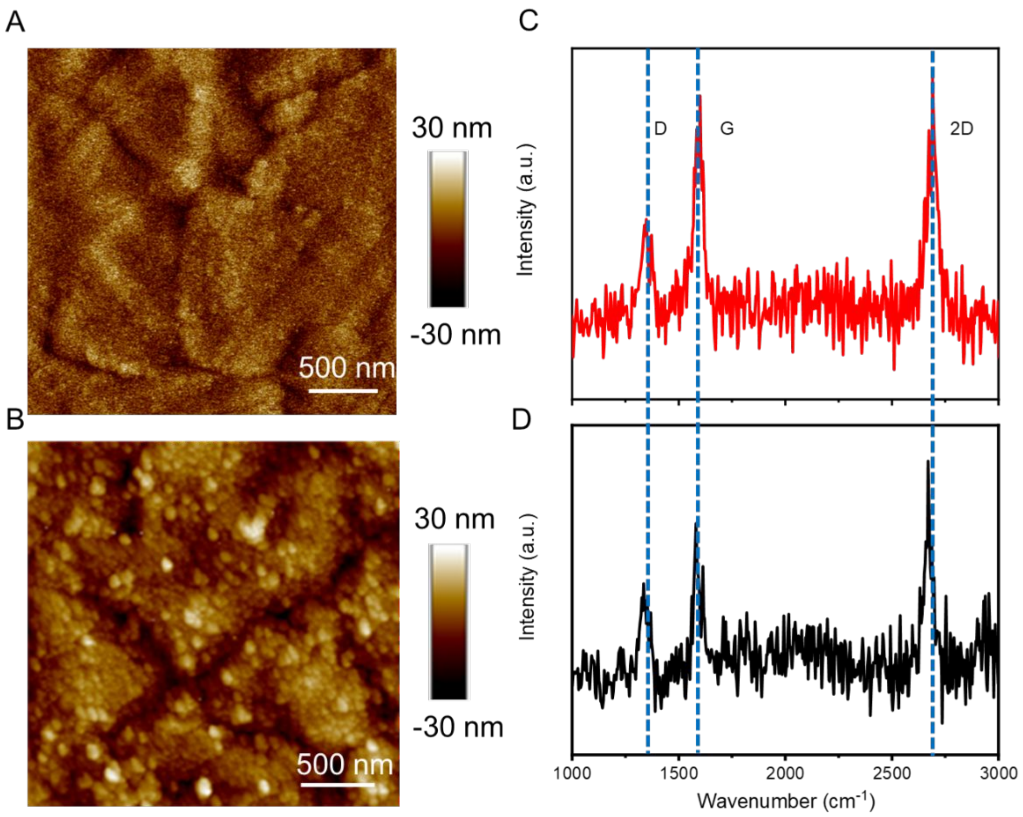

Figure S6: AFM images of a blank $Si/SiO_2/Ni/Cu//Graphene$ substrate (A) and a $Si/SiO_2/Ni/Cu//Graphene//3000Fe$ Aftn-AA substrate (B) Raman spectra of a blank Si/Ni/Cu//Graphene substrate (C) and a $Si/SiO_2/Ni/Cu//Graphene//3000Fe$ Aftn-AA substrate (D). The D, G and 2D peaks are highlighted with blue dashed lines.

Section S12. Temperature-dependent CT studies

Temperature-dependent CT studies for Aftn-AA immobilised on graphene were carried out in a Lakeshore CRX-VF probe station at intervals of 10 K in the range of $T = 250 – 330$ K under vacuum (1×10^{-5} mbar).

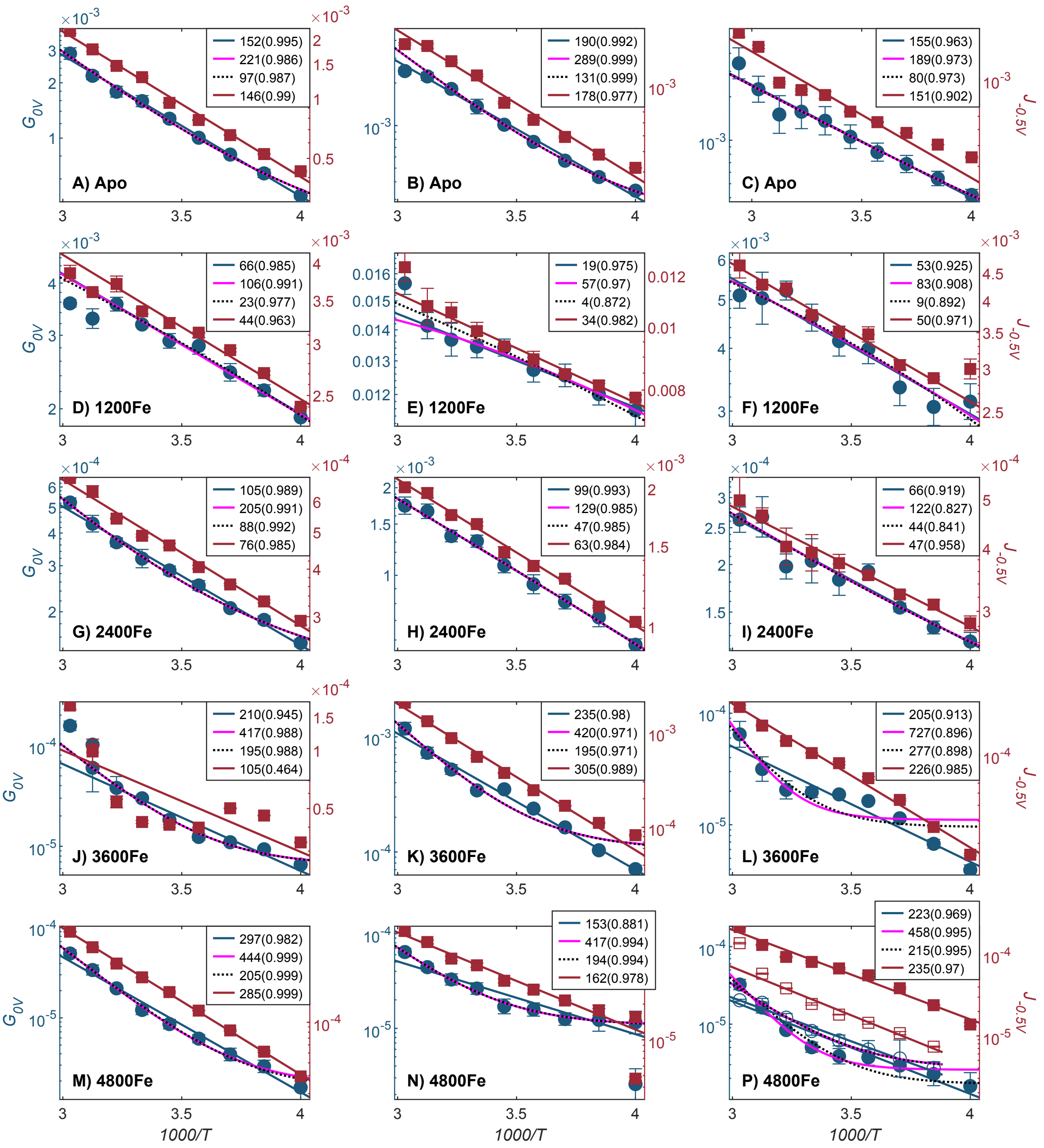

Figure S7: Arrhenius plots for near-zero conductance, G_{0V} (blue circles, left Y-axis) and current density at -0.5V, $J_{-0.5}$ (red squares, right-Y-axis) using a semi-log Y-scale against reciprocal temperature $(1000/T)$ for all measured junctions. Each row shows three repeats for the same Fe loading (see labels); the selected set presented in the main-text is shown on the left-most column. The 4800Fe loading was measured 4 times and therefore panel (P) includes an additional data set (hollow symbols). G_{0V} is the slope of current density w.r.t. voltage between ±0.02V (~5 data points). $J_{-0.5}$ is the current density at largest negative voltage (-0.5V). Symbols are geometric mean of ~6 repeated

voltage loops at each temperature, and error-bars represent standard deviation of $\log G_{0V}$ / $\log J_{-0.5}$. Lines are fits to following equations, and legend specifies extract activation energy (E_a in meV) and regression coefficient (R^2) for each fit.

Red: Equation 1 of main text (simple J-Arrhenius);

Blue: Equation 4 of main text (simple G-Arrhenius)

Magenta: Equation 2 of main text (Fermi-Dirac for G).

Dotted black: Equation S1 of SI (hopping model); often overlaps magenta line.

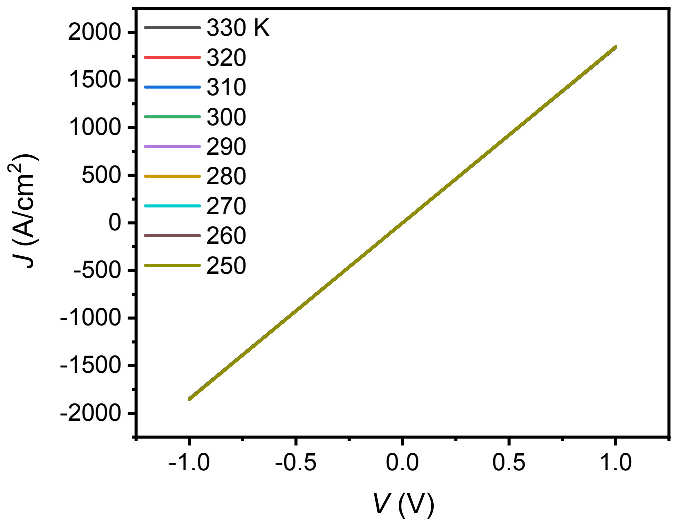

Figure S8: (A) $J(V,T)$ measurements on a blank Si/SiO_2/Ni/Cu//graphene substrate in the range of $T = 250 - 300$ K; the *J*-*V* curves are linear and independent of *T*.

Section S13. Graphene field-effect transistor (GFET) fabrication

In this work, the two terminal GFET device was fabricated using exfoliated graphene on Si/SiO_2 surface. The BLG was obtained by mechanical exfoliation with the standard scotch tape method and was transferred on highly doped Si/SiO_2. The source-drain electrodes

were patterned using e-beam lithography. The electrodes of Ti/Au of 5/100 nm were deposited by thermal evaporation. After the fabrication of the electrodes, the device was vacuum annealed at 200 ˚C for 2 h. The device was immersed into an aqueous solution of 3000Fe Aftn-AA for 2 h. As mentioned before, the Aftn-AA adsorbs on the graphene surface mediated by the primary NH_2 group present in the Aftn-AA.

We characterised the device with and without Aftn-AA by using Raman spectroscopy and AFM. Figure S9A and B show the AFM of the BLG before and after the adsorption of the Aftn-AA confirming the formation of a dense monolayer of Aftn-AA. Figure S10C shows the Raman spectra of the graphene as fabricated and after Aftn-AA adsorption which confirm the presence of a dense monolayer of graphene on BLG. The FWHM of the 2*D* peak is 54 cm^{-1} and absence of *D* peak at 1200 cm^{-1} for both samples suggest the presence of BLG in the GFET with a low defect density. The ratio of I_{2D}/I_G peak changes from 0.58 to 1.08 after Aftn-AA adsorption indicating the adsorption of charged biomolecules on the top surface of BLG.

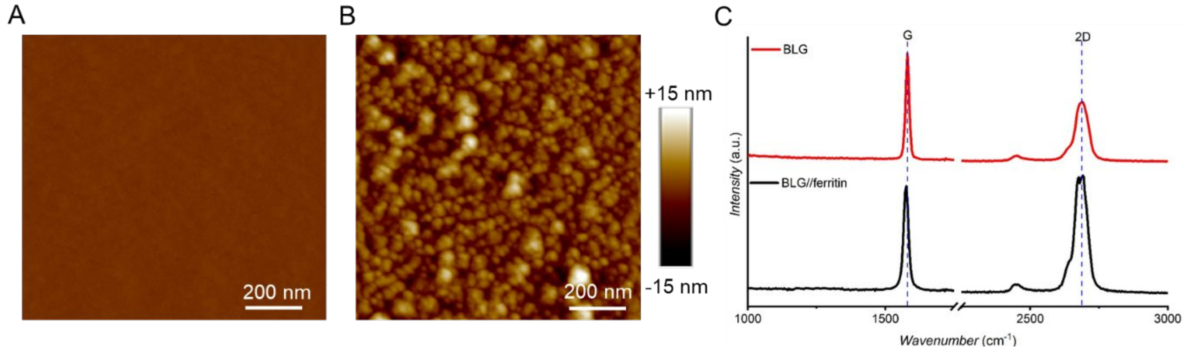

Figure S9: AFM images of the layer of BLG before (A) and after 3000Fe Aftn-AA adsorption. C) Raman spectra of BLG before and after 3000Fe Aftn-AA adsorption.

Section S14. Temperature-fitting and comparison of extracted parameters

G-V-T and *J-V-T* data are shown as blue and red symbols in Fig. S8 for 16 measured junctions. Fitting was done using MatLab fit.m function, with 'Trust-Region' algorithm and 'Bisquare' robust fitting (to under-weigh outliers).

In addition to the three possible temperature-dependencies mentioned in main text (Eqs. 1, 2, 4) we also fitted a slightly more complicated expression suggested by Asai & Tada[16] to describe super-exchange to hopping transition in molecular wires and specifically the cross-over to temperature-insensitive conductance:

$$G = G_{0\mathrm{K}} + G_{\infty}\exp\left[-\tanh\left(\frac{E_a}{k_\mathrm{B}T}\right)\right] \quad \text{(S1)}$$

Looking at Fig. S8 reveals minor differences in fit quality between the different models or using conductance (blue) or current (red) – observation per-se cannot signal out a specific temperature model. Therefore we wish to show that the trends in fitting parameters as shown in Fig. 4 of main-text are not an accidental result of using the Fermi-Dirac approach (Eq. 2, main text). Figure S10 compares the parameter, as extracted by the four alternative approaches presented in Fig. S8. A fifth option, shown only in Fig. S11 is:

$$G = {G_\infty}\Big/\left\{2k_\mathrm{B}T\left[1+\cosh\left({E_a}/{k_\mathrm{B}T}\right)\right]\right\} \quad \text{(S.2)}$$

This alternative is identical to Eq. 2, except of a missing saturation conductance ($G_{0K} \equiv 0$); it tests how influential the additional 3rd free parameter is compared to the change in exponential expression (e^x or $1 + \cosh x$).

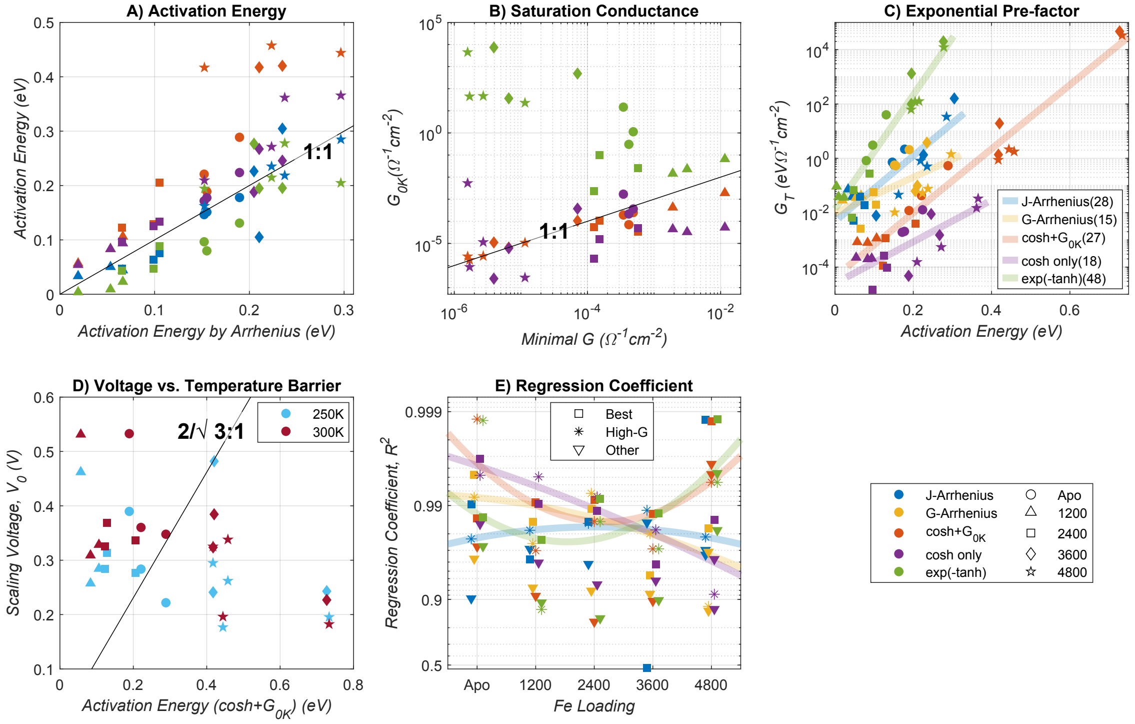

Figure S10: Consistency between CT parameters extracted by different methods, showing A) Activation energy, E_a; B) Saturation conductance, G_{0V}; C) Temperature pre-factor, G_∞; D) Scaling voltage, V_0; and E) Regression coefficient, R^2. To gain statistics, all 16 measured junctions are included and Fe-loading is marked by different shapes of symbols (see bottom-right legend), except of (E) where symbols (see legend) mark the columns of Fig. S8 (a rather arbitrary division). Colours (except of D) refer to temperature-analysis by:

- Blue: Current-based, standard Arrhenius (Eq. 1) – **2** fitting parameters;
- Yellow: Conductance-based, Arrhenius (Eq. 4) – **2** fitting parameters;
- Red: Fermi-Dirac view (Eq. 2) – **3** fitting parameters;
- Purple: No-intercept Fermi-Dirac (Eq. S2) – **2** fitting parameters;
- Green: Advanced hopping view (Eq. S1) – **3** fitting parameters;

The scaling voltage (D) is the only parameter that was extracted from analysing isothermal *I-V*, at the lowest measured temperature (250K, light blue) and room-temperature (300K,

maroon); it is plotted against activation energy extracted by Eq. 2 ('$\cosh + G_{0K}$' – red symbols of **A**).

The similar fitting quality is tested in Fig. S10E which compares the adjusted coefficient of determination, $\bar{R}^2$ (dimensionless) for the different fittings, where adjusted $\bar{R}^2$ is the standard R^2 corrected for the different number of free fitting parameters (2 or 3). Due to the wide scattering, trend lines (pale lines) are drawn using 2nd order polynomial smoothing. Triangles mark the 'poorly behaved' junctions – their $\bar{R}^2 \sim 0.9$ justifies their rejection; the fitting quality for *J*-Arrhenius (blue) was the least-scattered and least sensitive to loading (x-axis); in contrast, the fitting quality of $G - T$ across thick layers (3600-4800 Fe loading) improves significantly for three free parameters (red, green) compared to only two (yellow, purple) and vice versa for thin layers (Apo, 1200).

Fig. S10A shows activation energy values extracted by Eqs. 1, 2, S.1 and S.2 against a standard *G*-Arrhenius (Eq. 4). *J*-Arrhenius values (blue, Eq. 1) are very close to perfect replica (black line) implying negligible importance to choice of fitting $J - T$ or $G - T$. The other methods show consistent shifts relative to the black guide: use of $\cosh x$ adds a constant upward shift ($\sim 0.04e$V), using either two (purple) or three (red) free fitting parameters. The additional saturation conductance, $G_{0\mathrm{K}}$ significantly increases E_a only for high Fe loading (diamonds and stars), where ln $G vs. T^{-1}$ is clearly non-linear. The improved fit quality ($\bar{R}^2$, Fig. S10E) of Eq. 2 (red) vs. Eq. S.2 (purple) give credence to these exceptionally high E_a values. Beside this difference, the high correlation between $E_a$values extracted by different approaches supports the validity of extracted $E_a$values.

Considering the questionable need for a 3rd fitting parameter (G_{0K}), Fig. S10B plots G_{0K} against the minimal measured $G(0V)$ (log-log scale) of each junction, for the two models

which include it (Eq. 2 & Eq. S.1, red and green symbols, respectively). Purple symbols refer to Eq. S.2 (no G_{0K}) and mark the low 99% confidence level at the lowest T (250K). We see that G_{0K} values extracted by Eq. 2 (red) are generally close to minimal measured $G(0V)$ (black diagonal line) and generally above the lower-bound without G_{0K} (purple), indicating non-negligible values. The tested hopping model (Eq. S.1, green symbols) yields *negative* G_{0K} values (plotted values are: $-G_{0K}$), which we could not assign a physical meaning. For this reason, this explanation is not considered at all in the main text. Still, it is added here, first as a caution that fitting-quality alone could be deceiving, and second to substantiate the argument that a generic Arrhenius behaviour can be dressed by various exponential functions ($cosh$, $tanh$) keeping an identical fitting quality.

Fig. S10C reproduces Fig. 4C of main text: it plots temperature pre-factor, G_∞, against exponential factor, E_a, extracted by the various T-fitting-models for all 16 junctions. Shaded lines show a fit to exponential dependence of G_∞ on E_a, as reported in main text; the slope values are 15 – 50 eV^{-1}, as written in the legend. Interestingly, the exponential dependency holds even for two exceptionally high E_a values (red symbols, Eq. 2; omitted from panel A). This is solid support for the validity of the conclusion that the number of carriers increases with E_a, due to graphene's unique DOS (Fig. 5 of main text).

Finally, Fig. S10D examines an underlying assumption of Fermi-Dirac approach (Eq. 2), which identifies the activation energy, E_a, with the general CT energy barrier, ε_0 (red arrows in Fig. 5). In principle, ε_0, can also be extracted from the $J(V)$ dependency, especially using the scaling voltage, V_0, which measures the deviation of $J(V)$ from linearity (or Ohmic). Commonly, V_0 is defined as the voltage where $\frac{\mathrm{dlog}\,J}{\mathrm{dlog}\,V} = 2$ (also known as 'transition voltage', which is misleading as no transition is involved). Fig. S10D plots *voltage*-derived V_0At two extreme temperatures against *temperature*-derived E_a (using Eq. 2, referring to

0V). While both V_0 and E_a are spread around 0.3eV, V_0 has a much narrower spread than E_a (especially at room temperature) . More specifically, the black diagonal line marks: $V_0 = \frac{2}{\sqrt{3}} E_a$, dependency predicted assuming a Lorentzian transmission function and $E_a \equiv \varepsilon_0$ identity, as reported for perylene-based molecular junctions.[17] Fig. S10D indicates that Graphene // Ferritin junctions behave fundamentally different than this prediction. Specifically, the two outliers at $E_a \approx 0.7eV$ have lowest V_0 values contradicting the Lorentzian-expected positive, linear relation. Interestingly, assuming a Gaussian transmission function (cf. Lorentzian), does yield an inversed dependency ($V_0 \propto \frac{1}{E_a}$).[18,19] The data in Fig. S10D is too scattered to affirmatively identify the shape of the transmission function, except that the dependency is more complicated than a simplified Lorentzian-shaped transmission function.

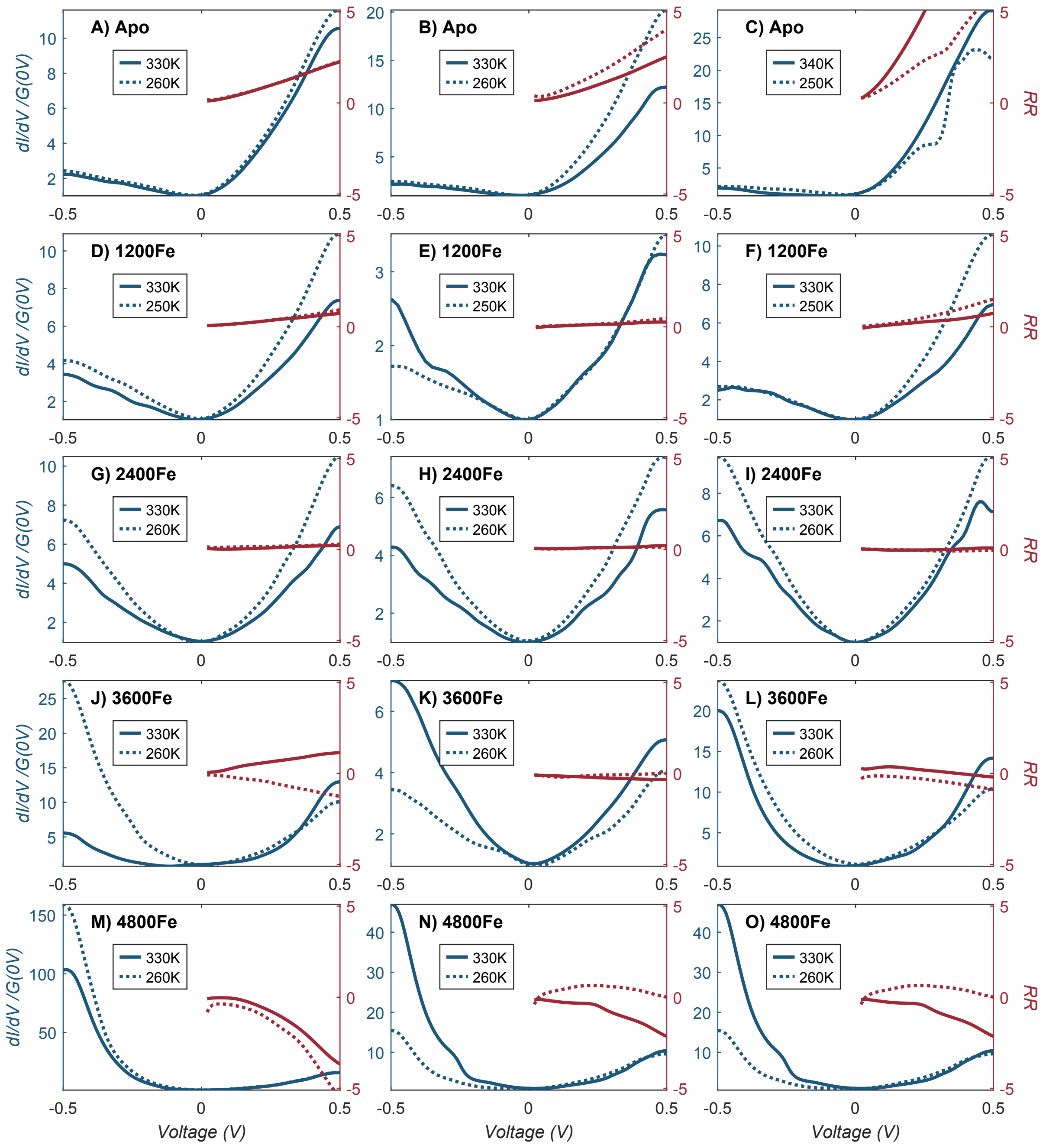

Figure S11: Transport asymmetry showing normalised conductance (left Y-axis, blue) and rectification ratio (RR, right Y-axis, red) for all measured junctions, at highest (solid line) and lowest (dotted line) measured temperatures, measured at a base pressure of 1×10^{-3} mbar. Each row shows three repeats for the same Fe loading (see labels); the selected set presented in the main-text is shown on the left-most column. Conductance is the first derivative (dI/dV) computed numerically, which is then normalised by its values at 0V (minimal value of all left Y-axis is 1). The rectification ratio is

defined as $RR_i = \frac{|I_i^+| - |I_i^-|}{min(|I_i^+|,|I_i^-|)}$, where subscript i refers to identical $\pm|V|$ values (negative RR implies larger absolute current at negative voltage).

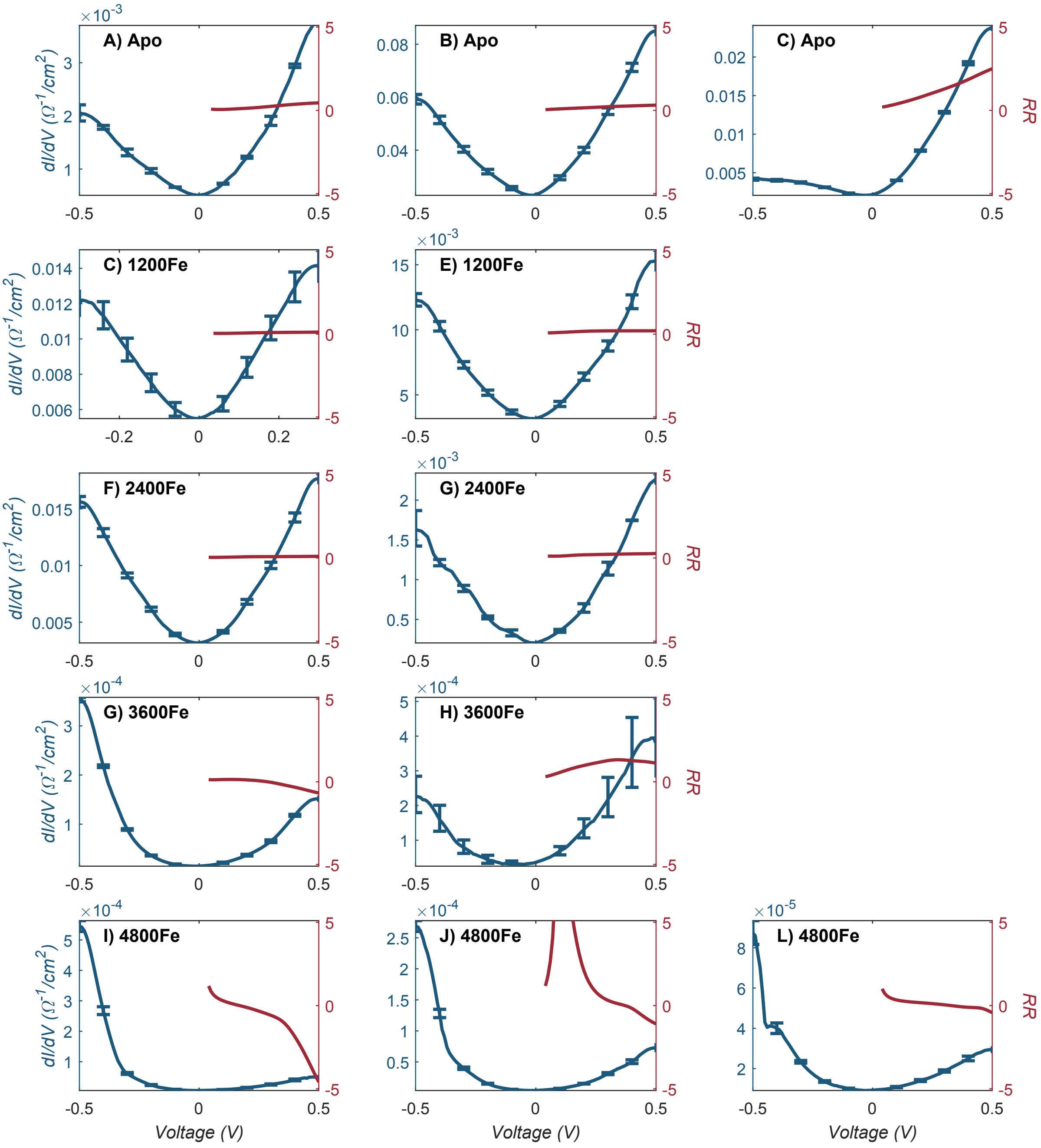

Figure S12: Transport asymmetry under ambient conditions: roughly: 298K one atmosphere and un-controlled humidity. Conductance values (left Y-axis) are direct values (no normalisation) and are

averaged over 40-100 repeats, with error bars marking standard deviation. RR values are computed as in Fig. S12.

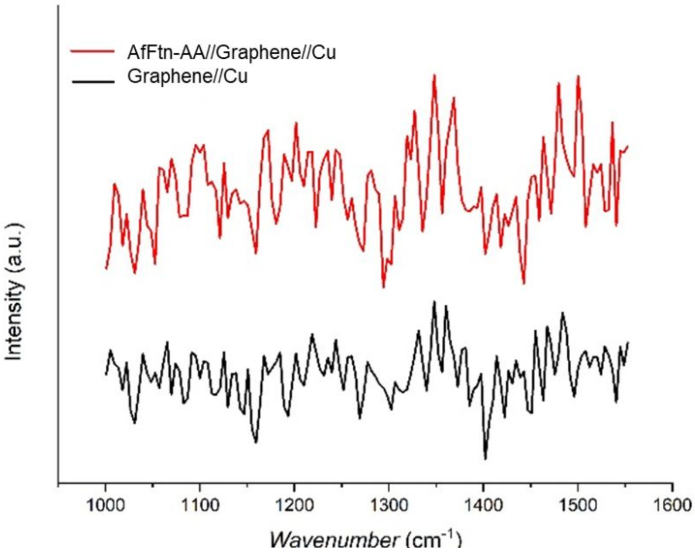

Figure S13: Raman spectra of the graphene before and after adsorption of the AfFtn-AA. No changes in the intensity of the peak at 1350 cm^{-1} is observed, suggesting that AfFtn-AA adsorption does not increase the number of defects in graphene.

References:

(1) Kumar, K. S.; Pasula, R. R.; Lim, S.; Nijhuis, C. A. Long-Range Tunneling Processes across Ferritin-Based Junctions. *Adv. Mater.* **2016**, *28* (9), 1824–1830.

(2) Sana, B.; Johnson, E.; Sheah, K.; Poh, C. L.; Lim, S. Iron-Based Ferritin Nanocore as a Contrast Agent. *Biointerphases* **2010**, *5* (3), FA48–FA52.

(3) Liu, X.; Jin, W.; Theil, E. C. Opening Protein Pores with Chaotropes Enhances Fe Reduction and Chelation of Fe from the Ferritin Biomineral. *Proc. Natl. Acad. Sci.* **2003**, *100* (7), 3653–3658.

(4) Li, X.; Cai, W.; An, J.; Kim, S.; Nah, J.; Yang, D.; Piner, R.; Velamakanni, A.; Jung, I.; Tutuc, E.; Banerjee, S. K.; Colombo, L.; Ruoff, R. S. Large-Area Synthesis of High-Quality and Uniform Graphene Films on Copper Foils. *Science (80-.).* **2009**, *324* (5932), 1312–1314.

(5) Chen, X.; Salim, T.; Zhang, Z.; Yu, X.; Volkova, I.; Nijhuis, C. A. Large Increase in the Dielectric Constant and Partial Loss of Coherence Increases Tunneling Rates across Molecular Wires. *ACS Appl. Mater. Interfaces* **2020**, *12* (40), 45111–45121.

(6) Martin, K. C.; Villano, S. M.; McCurdy, P. R.; Zapien, D. C. Studies of Electrochemically Transformed Ferritin Adsorbed at Tin-Doped Indium Oxide Electrodes Using X-Ray Photoelectron Spectroscopy. *Langmuir* **2003**, *19* (14), 5808–5812.

(7) Gálvez, N.; Fernández, B.; Sánchez, P.; Cuesta, R.; Ceolín, M.; Clemente-León, M.; Trasobares, S.; López-Haro, M.; Calvino, J. J.; Stéphan, O.; Domínguez-Vera, J. M.; Gálvez, N.; Fernández, B.; Sánchez, P.; Cuesta, R.; Ceolín, M.; Clemente-León, M.; Trasobares, S.; López-Haro, M.; Calvino, J. J.; Stéphan, O.; Domínguez-Vera, J. M. Comparative Structural and Chemical Studies of Ferritin Cores with Gradual Removal of Their Iron Contents. *J. Am. Chem. Soc.* **2008**, *130* (25), 8062–8068.

(8) Ilari, A.; Stefanini, S.; Chiancone, E.; Tsernoglou, D. The Dodecameric Ferritin from Listeria Innocua Contains a Novel Intersubunit Iron-Binding Site. *Nat. Struct. Biol.* **2000**, *7* (1), 38–43.

(9) Nerngchamnong, N.; Yuan, L.; Qi, D.-C.; Li, J.; Thompson, D.; Nijhuis, C. A. The Role of van Der Waals Forces in the Performance of Molecular Diodes. *Nat. Nanotechnol.* **2013**, *8* (2), 113–118.

(10) Wan, A.; Jiang, L.; Sangeeth, C. S. S. S.; Nijhuis, C. A. Reversible Soft Top-Contacts to Yield Molecular Junctions with Precise and Reproducible Electrical Characteristics. *Adv. Funct. Mater.* **2014**, *24* (28), 4442–4456.

(11) Dong, Y.; Guo, S.; Mao, H.; Xu, C.; Xie, Y.; Cheng, C.; Mao, X.; Deng, J.; Pan, G.; Sun, J. The Growth of Graphene on Ni–Cu Alloy Thin Films at a Low Temperature and Its Carbon Diffusion Mechanism. *Nanomaterials* **2019**, *9* (11), 1633.

(12) Yang, G.; Li, L.; Lee, W. B.; Ng, M. C. Structure of Graphene and Its Disorders: A Review. *Sci. Technol. Adv. Mater.* **2018**, *19* (1), 613–648.

(13) Tian, W.; Li, W.; Yu, W.; Liu, X. A Review on Lattice Defects in Graphene: Types, Generation, Effects and Regulation. *Micromachines* **2017**, *8* (5), 163.

(14) Vicarelli, L.; Heerema, S. J.; Dekker, C.; Zandbergen, H. W. Controlling Defects in Graphene for Optimising the Electrical Properties of Graphene Nanodevices. *ACS Nano* **2015**, *9* (4), 3428–3435.

(15) Das, A.; Pisana, S.; Chakraborty, B.; Piscanec, S.; Saha, S. K.; Waghmare, U. V.; Novoselov, K. S.; Krishnamurthy, H. R.; Geim, A. K.; Ferrari, A. C.; Sood, A. K. Monitoring Dopants by Raman Scattering in an Electrochemically Top-Gated Graphene Transistor. *Nat. Nanotechnol.*

2008, *3* (4), 210–215.

(16) Lee, S. K.; Yamada, R.; Tanaka, S.; Chang, G. S.; Asai, Y.; Tada, H. Universal Temperature Crossover Behavior of Electrical Conductance in a Single Oligothiophene Molecular Wire. *ACS Nano* **2012**, *6* (6), 5078–5082.

(17) Smith, C. E.; Xie, Z.; Bâldea, I.; Frisbie, C. D. Work Function and Temperature Dependence of Electron Tunneling through an N-Type Perylene Diimide Molecular Junction with Isocyanide Surface Linkers. *Nanoscale* **2018**, *10* (3), 964–975.

(18) Vilan, A.; Cahen, D.; Kraisler, E. Rethinking Transition Voltage Spectroscopy within a Generic Taylor Expansion View. *ACS Nano* **2013**, *7* (1), 695–706.

(19) Bâldea, I. Important Issues Facing Model-Based Approaches to Tunneling Transport in Molecular Junctions. *Phys. Chem. Chem. Phys.* **2015**, *17* (31), 20217–20230.
